# Characterizing Mutational Signatures in Human Cancer Cell Lines Reveals Episodic APOBEC Mutagenesis

**DOI:** 10.1016/j.cell.2019.02.012

**Published:** 2019-03-07

**Authors:** Mia Petljak, Ludmil B. Alexandrov, Jonathan S. Brammeld, Stacey Price, David C. Wedge, Sebastian Grossmann, Kevin J. Dawson, Young Seok Ju, Francesco Iorio, Jose M.C. Tubio, Ching Chiek Koh, Ilias Georgakopoulos-Soares, Bernardo Rodríguez–Martín, Burçak Otlu, Sarah O’Meara, Adam P. Butler, Andrew Menzies, Shriram G. Bhosle, Keiran Raine, David R. Jones, Jon W. Teague, Kathryn Beal, Calli Latimer, Laura O’Neill, Jorge Zamora, Elizabeth Anderson, Nikita Patel, Mark Maddison, Bee Ling Ng, Jennifer Graham, Mathew J. Garnett, Ultan McDermott, Serena Nik-Zainal, Peter J. Campbell, Michael R. Stratton

**Affiliations:** 1 Cancer, Ageing and Somatic Mutation, Wellcome Sanger Institute, Hinxton, Cambridgeshire CB10 1SA, UK; 2 Department of Cellular and Molecular Medicine and Department of Bioengineering, Moores Cancer Center, University of California, San Diego, La Jolla, CA 92093, USA; 3 Oxford Big Data Institute, Old Road Campus, Oxford OX3 7LF, UK; 4 Oxford NIHR Biomedical Research Centre, Oxford, OX4 2PG, UK; 5 Graduate School of Medical Science and Engineering, Korea Advanced Institute of Science and Technology, Daejeon 305-701, Republic of Korea; 6 European Molecular Biology Laboratory - European Bioinformatics Institute, Hinxton, Cambridgeshire CB10 1SA, UK; 7 Mobile Genomes and Disease, Molecular Medicine and Chronic Diseases Centre (CIMUS), Universidade de Santiago de Compostela, Santiago de Compostela 15706, Spain; 8 Department of Zoology, Genetics and Physical Anthropology, Universidade de Santiago de Compostela, Santiago de Compostela 15706, Spain; 9 The Biomedical Research Centre (CINBIO), Universidade de Vigo, Vigo 36310, Spain; 10 Cytometry Core Facility, Wellcome Sanger Institute, Hinxton, Cambridgeshire CB10 1SA, UK; 11 Department of Medical Genetics, The Clinical School, University of Cambridge, Cambridge, CB2 0QQ, UK

**Keywords:** mutational signatures, cancer cell lines, xenografts, APOBEC deaminases, episodic mutagenesis

## Abstract

Multiple signatures of somatic mutations have been identified in cancer genomes. Exome sequences of 1,001 human cancer cell lines and 577 xenografts revealed most common mutational signatures, indicating past activity of the underlying processes, usually in appropriate cancer types. To investigate ongoing patterns of mutational-signature generation, cell lines were cultured for extended periods and subsequently DNA sequenced. Signatures of discontinued exposures, including tobacco smoke and ultraviolet light, were not generated *in vitro*. Signatures of normal and defective DNA repair and replication continued to be generated at roughly stable mutation rates. Signatures of APOBEC cytidine deaminase DNA-editing exhibited substantial fluctuations in mutation rate over time with episodic bursts of mutations. The initiating factors for the bursts are unclear, although retrotransposon mobilization may contribute. The examined cell lines constitute a resource of live experimental models of mutational processes, which potentially retain patterns of activity and regulation operative in primary human cancers.

## Highlights

• Annotation of mutational signatures across 1,001 cancer cell lines and 577 PDXs
• Activities of mutational processes determined over time in cancer cell lines
• APOBEC-associated mutagenesis is often ongoing and can be episodic
• Detection of mutational signatures by single-cell sequencing

## Graphical Abstract

**Figure:**
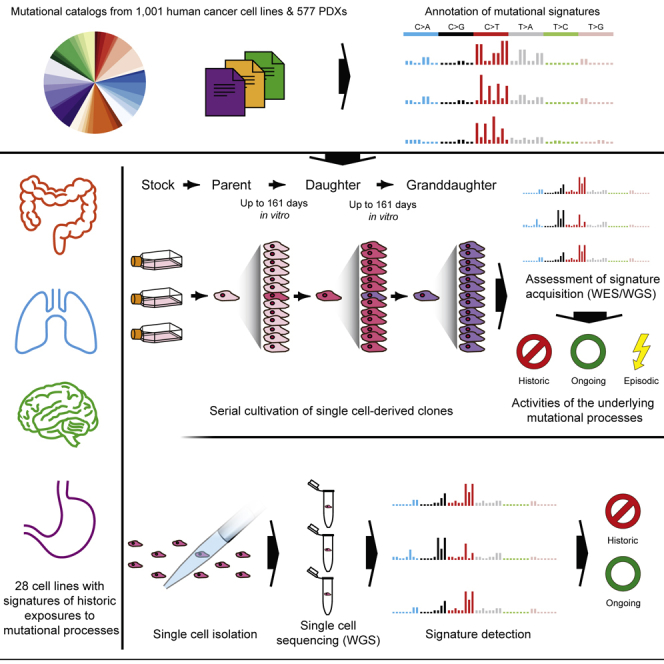

## Teaser

An analysis of 1,001 human cancer cell lines and 577 xenografts shows that mutagenesis associated with the cytodine deaminase APOBEC occurs in episodic bursts in contrast to mutation signatures associated with DNA replication and repair.

## Introduction

Each mutational process operative in a cell leaves a mutational signature imprinted on its genome (Stratton et al., 2009). Using mathematical approaches applied to thousands of catalogs of somatic mutations from the range of human cancer types (Alexandrov et al., 2013b), more than 40 base substitution and ten genome rearrangement mutational signatures have thus far been identified (Alexandrov et al., 2018, Alexandrov et al., 2013a, Li et al., 2017, Nik-Zainal et al., 2016). There is currently insight into the mutational processes underlying about half of these signatures (Helleday et al., 2014, Petljak and Alexandrov, 2016). However, many questions remain pertaining to the biology of their underlying mechanisms, which require experimental models to be addressed.

The somatic mutational catalog of a cancer genome is the aggregate of mutations that has been generated by multiple mutational processes active at any point during the cell lineage from the fertilized egg to the cancer cell (Stratton et al., 2009). Some mutational processes may operate continuously and for the full duration of the cell lineage, as proposed for the processes underlying signatures 1 and 5, which are ubiquitous among cancer types and are found in normal cells (Alexandrov et al., 2015, Blokzijl et al., 2016). Others may operate over only part of the lineage and may no longer be active when the cancer is sampled, for example, exposures to tobacco smoke and ultraviolet light. Comparisons of mutations generated during different phases of the evolution of individual human cancers *in vivo* suggest that some mutational processes show varying degrees of activity over time (Gerstung et al., 2017, McGranahan et al., 2015, Nik-Zainal et al., 2012a).

To provide a resource for experimental investigation of the biological mechanisms underlying the repertoire of mutational signatures, we first annotated mutational signatures on sets of publicly available models, including 1,001 immortal human cell lines (COSMIC Cell Line Project) and 577 patient-derived xenografts (PDXs; NCI Patient-Derived Models Repository) derived from a broad spectrum of cancer types. The panel includes most widely used models in cancer research and therapeutics testing and is being extensively characterized genomically, transcriptomally, epigenomically, and for biological responses to therapeutics (Garnett et al., 2012, Iorio et al., 2016).

We subsequently used a subset of the cancer cell lines to experimentally assess whether mutational processes underlying mutational signatures continue to be active during *in vitro* culture and to characterize their temporal patterns of activity. Cell lines continuing to acquire mutational signatures represent informative models for future investigation of their underlying mechanisms.

## Results

### Mutational Signatures in Cancer Cell Lines and PDX Models

**Figure 1:**
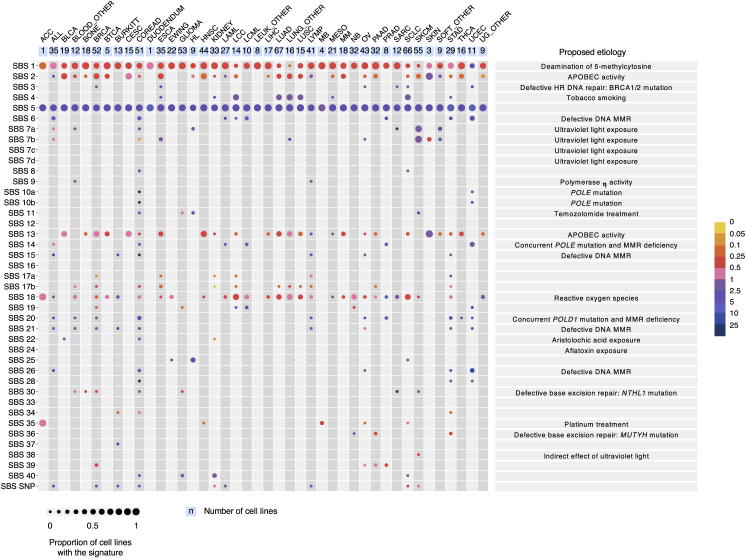
Mutational Signatures in 1,001 Human Cancer Cell Lines Cancer cell line classes are ordered alphabetically as columns, and mutational signatures are displayed as rows. The cell line classification was modified from the COSMIC Cell Line Project (see Table S2). For patterns of mutational signatures, see Figure S1. The figure format follows the annotation of mutational signatures across a large set of primary human cancers done previously (Alexandrov et al., 2018). We thank the members of the International Cancer Genome Consortium (ICGC) Pan-Cancer Analysis of Whole Genomes (PCAWG) project for the figure design.

**Figure S1:**
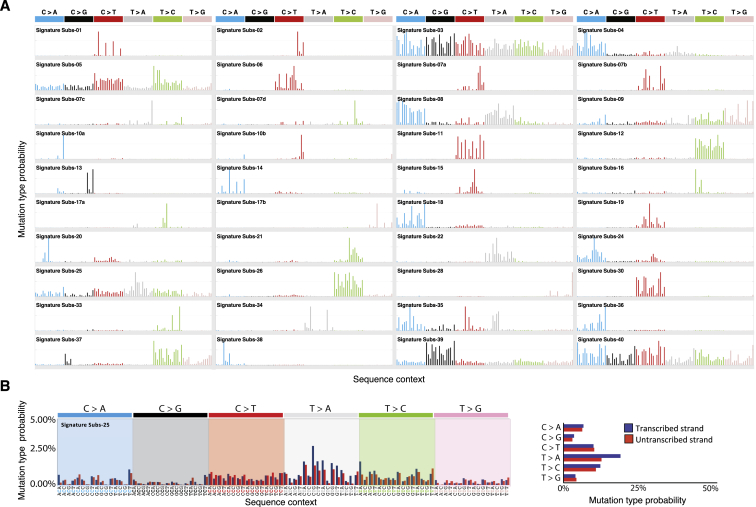
Core Set of the Annotated Mutational Signatures, Related to Figures 1, 3, 5, and 6 (A) The core set of the mutational signatures, including the Platinum set of the PCAWG signatures and SBS25 discovered in Hodgkin’s lymphoma cell lines. Signatures are displayed according to the alphabetical 96-substitution classification on horizontal axes, defined by the six color-coded substitution types and sequence context immediately 5′ and 3′ to the mutated base axes (as per panel B). Vertical axes differ between individual signatures for visualization of their patterns (numerical patterns in Table S1) and indicate the percentage of mutations attributed to specific mutation types, adjusted to genome-wide trinucleotide frequencies. We thank PCAWG Mutational Signatures Working Group for the figure. (B) Transcriptional strand bias for SBS25. The mutational signature is displayed according to the 192-subsitution classification, incorporating the six substitution types in color-coded panels, the sequence context immediately 5′ and 3′ to the mutated base and whether the mutated base (in pyrimidine context) is on the transcribed (blue bars) or untranscribed (red bars) strand.

The presence and relative contributions of single base substitution signatures (SBSs) were determined in each of 1,001 cancer cell lines (Figure 1; Table S3) and 577 PDX models (Table S3), derived from more than 40 cancer types using previously generated whole-exome DNA sequences (STAR Methods; signature patterns in Figure S1 and Table S1). The analysis revealed a novel signature of unknown origin in Hodgkin’s lymphoma cell lines characterized by T>A base substitutions (termed SBS25; Figures 1 and S1). During manuscript revision, attribution of a more limited set of mutational signatures to the same set of cancer cell lines was reported (Jarvis et al., 2018).

The majority of the base substitution signatures observed in primary cancers (Alexandrov et al., 2018) were found in the examined cell line and PDX models (Figure 1; Table S3). These included signatures of exogenous environmental exposures such as SBS4, caused by tobacco-smoke exposure, in lung cancers; SBS7a-b and SBS38, caused by ultraviolet light, in melanoma models; SBS11, likely caused by temozolomide treatment, in melanoma and glioma cell lines; SBS22, caused by aristolochic acid (Poon et al., 2013), in a bladder cancer cell line; and SBS35, associated with platinum compound chemotherapy (Boot et al., 2018), in ovarian and sarcoma models.

Signatures associated with mutational processes of endogenous origins were also found, including SBS2 and SBS13, associated with APOBEC (apolipoprotein B mRNA editing enzyme, catalytic polypeptide-like) cytidine deaminase DNA-editing activity (Nik-Zainal et al., 2012a), in cell lines and PDXs from breast, bladder, head and neck, cervix, lung, esophageal, and non-melanoma skin carcinomas; signatures associated with microsatellite instability (MSI) due to defective DNA mismatch repair (MMR) (SBS6, SBS15, SBS21, and SBS26) and due to a concurrent loss of MMR and proofreading functions of polymerases epsilon (POLE; SBS14) or polymerase delta (POLD; SBS20) (Haradhvala et al., 2018), in colorectal, gastric, and endometrial models; SBS10a-b, due to mutations in *POLE*, in colorectal, endometrial, and stomach models; SBS36, associated with defective base excision repair and *MUTYH* mutations (Pilati et al., 2017, Viel et al., 2017), in pancreatic cell lines; and SBS3, associated with defective homologous recombination-based double-strand break (HR-DSB) DNA repair often due to BRCA1 or BRCA2 inactivation (Nik-Zainal et al., 2012a), in breast, ovarian, sarcoma, and esophageal models.

Finally, signatures of uncertain and speculative origins (Alexandrov et al., 2018) were also observed, including SBS17a-b in gastric and esophageal models; SBS18, potentially due to reactive-oxygen-species-induced DNA damage, in neuroblastoma cell lines; SBS9, which might result from aberrant processing of AID-induced cytidine deamination by polymerase η, in lymphoma cell lines; and SBS28, SBS34, SBS39, and SBS40, which were found mainly in the cancer types in which they had been previously reported. SBS1 (associated with deamination of 5-methyl cytosine) and SBS5 (of unknown origin) are ubiquitous among cancer types (Alexandrov et al., 2015) and were present in most cancer cell lines and PDXs. However, some SBS1 and SBS5 mutations are likely attributable to residual germline variants, which remain because of the non-availability of normal DNAs from the same individuals for most cancer cell lines (STAR Methods) and which are also constituted of these two signatures (Rahbari et al., 2016).

A small subset of signatures was absent from the examined datasets (SBS7c, SBS12, SBS16, SBS24) or found less often than expected (e.g., SBS3) (Alexandrov et al., 2018). These may be due to the small numbers of somatic mutations in exome sequences, the small numbers of mutations some signatures contribute to individual cancers, the obscuring presence of residual germline variants, the relatively featureless profiles of some signatures that may be more difficult to detect, and/or the genuine absence of the signatures (Alexandrov et al., 2013b). Some signatures were detected in a small proportion of models from cancer classes in which they have not been previously reported (Alexandrov et al., 2018). Such instances likely reflect past misclassification, past cross-contamination, or minor misattribution of mutational signatures.

### Investigating Continuing Mutagenesis in Cancer Cell Lines

To investigate the patterns of activity of mutational processes underlying a wide range of signatures, we selected 28 cell lines derived from cancers of the breast, colorectum, uterus, lung, stomach, cervix, ovary, head and neck, skin (melanoma and squamous), white blood cells (B cell lymphoma and leukemia), and neuroblastoma (Figure 3A). One or more of these had high contributions from mutational signatures of tobacco smoke (SBS4); ultraviolet light (SBS7a-d); aberrant APOBEC cytidine deaminase activity (SBS2 and SBS13); defective DNA MMR with MSI (SBS6, SBS15, SBS21, SBS26); concurrent loss of MMR and proofreading functions in POLD (SBS20) and POLE (SBS14); aberrant POLE activity (SBS10a-b); deficiency of HR-DSB repair (SBS3); and signatures of uncertain origin including SBS17a-b (frequently found in esophageal and gastric cancers), SBS18 (found commonly in neuroblastoma), and SBS28 (common in colorectal and endometrial cancers with mutations in *POLE*). SBS1 and SBS5 were detected in most cell lines.

**Figure 2:**
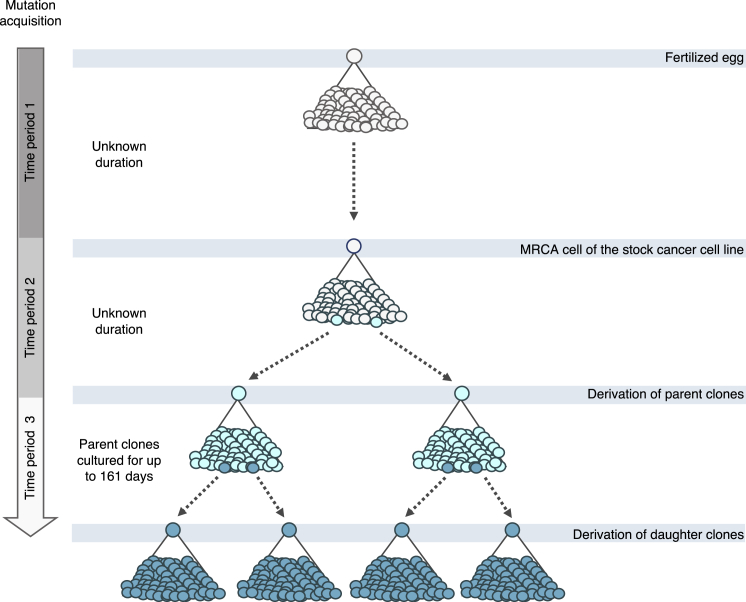
Tracking Mutation Acquisition in a Cancer Cell Line over Time Sequence from the stock cell line captures mostly clonal somatic mutations acquired from the fertilized egg to the establishment of the most recent common ancestor cell (MRCA) of the cell line population (*period 1*) and residual germline variation due to the non-availability of the reference normal DNA from the same individuals. The somatic mutations were acquired during an unknown period of time, predominantly *in vivo* during the life of the cancer patient, although a small proportion may have been acquired *in vitro* if establishment of the MRCA cell occurred in culture. Sequences from the single-cell-derived parent clones include the same set of mutations, with an addition of mutations acquired between the establishment of the MRCA cell of the stock cell line and isolation of the single parent cells (*period 2*). Duration of this period is unknown as it depends on timing of the establishment of the MRCA cell and hence may include an *in vivo* time frame. Mutations generated during this period were revealed by subtracting sequences of stock cell lines from those of parent clones. Sequences from single-cell-derived daughter clones include the mutations from their parent clones and, in addition, mutations acquired *in vitro* during the defined cultivation time frames spanning the two single-cell isolation events (up to 161 days, *period 3*). Subtraction of the sequences of parent clones from those of daughter clones therefore reveals mutations acquired during the examined *in vitro* periods. Clones in Figure 3, Figure 4, Figure 5 follow the outlined experimental design, but the numbers of obtained clones and generations may vary.

**Figure 3:**
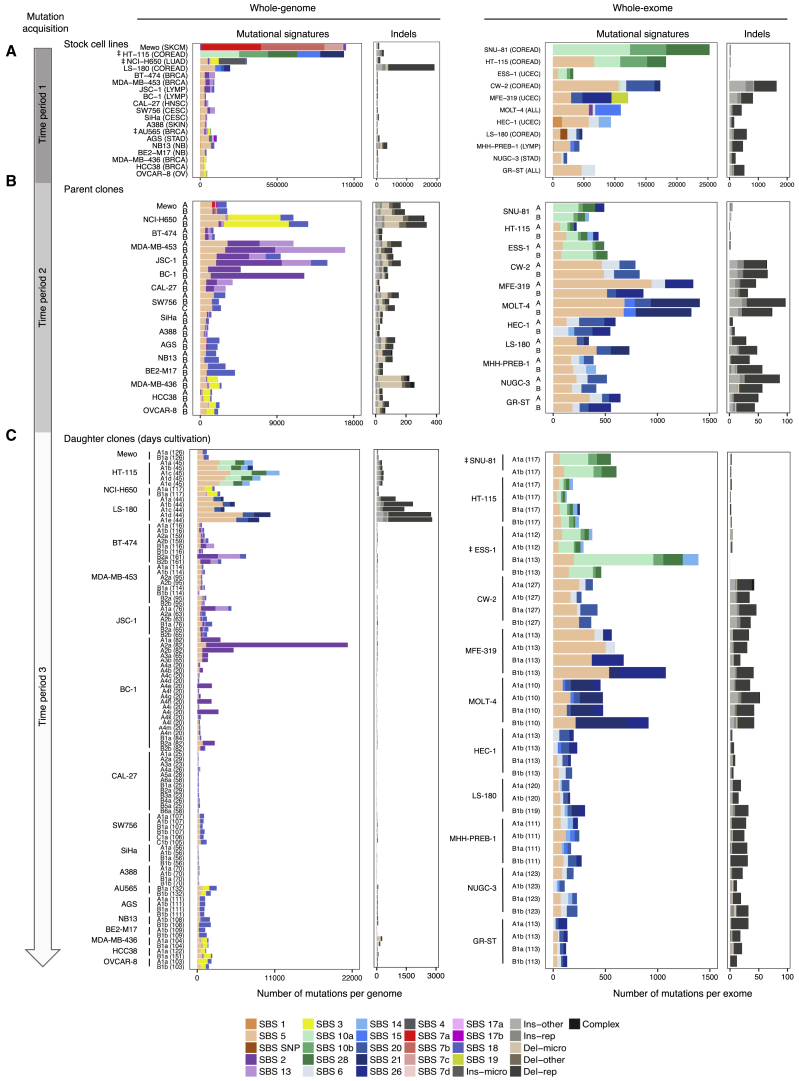
Activities of Mutational Processes in Human Cancer Cells (A–C) Bars represent the numbers of base substitutions attributed to mutational signatures (patterns in Figure S1) and indels in stock cell lines (A; cancer type abbreviations in Table S2) and their respective parent (B) and daughter or granddaughter clones (C), which were acquired during the indicated time frames following the experimental design in Figure 2. Daughter clones were cultivated for the numbers of days indicated in brackets. Mutational signatures are ordered and colored according to the associated etiologies. Ins/del - rep/micro/other, small insertions/deletions at repetitive regions, microhomology-mediated or other; complex, complex indels. ‡ Only single parent clones from HT-115, LS-180, and AU565 cell lines were subject to whole-genome sequencing, and their sequences were used as proxies for the mutational catalogs of the corresponding stock cell lines (STAR Methods). The high number of mutations in ESS-1 B1a clone is likely due to its establishment from two cells (Figure S7). Daughters were not successfully established from SNU-81_B parent clone.

One or more single-cell-derived subclones were established from the stock cultures of each of the 28 cancer cell lines (STAR Methods; Figure 2). These subclones of the stock culture were termed “parent” clones. Parent clones were then propagated in culture for up to 161 days (Table S2). Following this period of cultivation, a further round of subcloning was carried out on the cell population from each parent clone, and one or more single-cell subclones were derived (Figure 2). These single-cell subclones of the parent clone were termed “daughter” clones. Daughter clones were expanded in culture to generate a population of cells from which sufficient DNA for further analysis could be obtained. DNAs extracted from parent and daughter clones were whole-exome and/or whole-genome sequenced, and mutations were called (STAR Methods; Figure S7). Subtraction of mutations present in parent clones from those in related daughter clones yielded the mutation sets acquired predominantly during the periods of *in vitro* propagation between the two subcloning events (STAR Methods). Subtraction of mutations present in the stock cell lines from mutations in their corresponding parent clones (or in some instances, subtraction of mutations shared by two parent lines) revealed mutations acquired mostly between the establishment of the most recent common ancestor cell of the stock cell line and isolation of the single parent cells (STAR Methods). The signature profile of 100 whole-genome- and 41 whole-exome-sequenced daughter clones alongside their corresponding 58 parent clones was then generated (Figure 3; Table S3; STAR Methods).

Certain mutational signatures present in stock cell lines were not generated during *in vitro* culture of their descendant clones (Figure 3; Table S3). These included SBS4 and SBS7 due to tobacco smoke and ultraviolet light, respectively, to which the examined lung cancer (NCI-H650) and melanoma (Mewo) cell lines were not exposed during *in vitro* culture. In addition, SBS17a-b did not continue to be acquired *in vitro* in the stomach (AGS) cell line overwhelmed with these signatures. All other signatures present in stock cell lines continued to be generated during culture of descendant clones from at least some cell lines.

**Figure S2:**
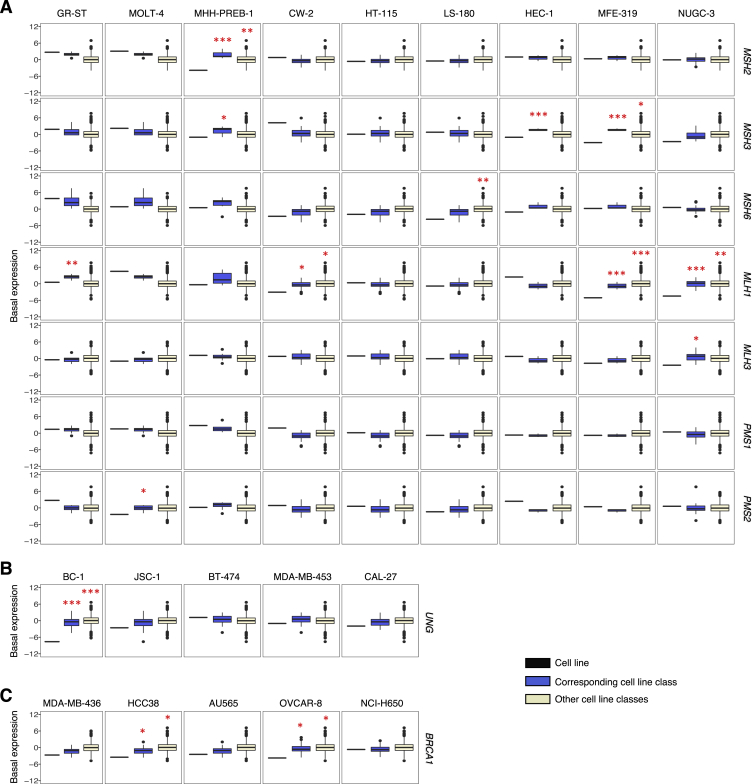
Expression of Genes Previously Associated with Mutational Signatures in Examined Cancer Cell Lines, Related to Figures 3 and 4 Each panel compares normalized basal expression of indicated genes, between the examined cell line (black) indicated on the top and cell lines from the 1,001 panel, from matching (blue) or other (beige) cell line classes as per their COSMIC classification (Table S2). P values (one-tailed; ^∗^p < 0.05, ^∗∗^p < 0.01, ^∗∗∗^p < 0.001) correspond to the computed z-scores indicating the deviation of the mean expression of the gene in the examined cell line from the groups used in comparisons. (A) Expression of the mismatch repair genes in cell lines with MSI-associated signatures (SBS6, SBS14, SBS15, SBS20, SBS21, SBS26). Cell lines classified as high or low in microsatellite instability (Iorio et al., 2016) were excluded from the control panels. (B) Expression of *UNG* in cell lines with APOBEC-associated SBS2 and SBS13. (C) Expression of *BRCA1* in cell lines with SBS3, associated with defective activity of the homologous-recombination-based double-strand break repair.

Multiple mutational signatures have previously been associated with defective mismatch repair (SBS6, SBS14, SBS15, SBS20, SBS21, SBS26) (Alexandrov et al., 2018). In some cell lines, the particular signature(s) present in the stock converted to different defective MMR signature(s) during *in vitro* culture (e.g., acute lymphocytic leukemia MOLT-4) or remained roughly stable (e.g., colorectal cancer CW-2) (Figure 3; Table S3). In others, certain signatures appeared to be present in the stock cell line but were absent from all or some clones and vice versa. However, visual inspection of mutation spectra indicated that this was likely due to misattribution of mutations to other MMR-deficiency signatures and that all cell lines with defective MMR (Table S4; Figure S2) continued to generate a subset of the corresponding signatures alongside the large numbers of small indels at short nucleotide repeats typical of this repair deficiency (Figure 3).

The colorectal (SNU-81 and HT-115) and endometrial (ESS-1) cancer cell lines with mutations in *POLE* (Table S4) continued to generate the associated base substitution signatures. However, the relative contribution of SBS10b (composed predominantly of C>T mutations) compared to signature SBS10a (composed predominantly of C>A mutations) diminished markedly *in vitro* (Figure 3). Furthermore, signature 28, often found in cancers with mutations in *POLE* (Alexandrov et al., 2018), continued to be generated in all of these cell lines but not in the examined stomach cancer cell line (AGS) (Figure 3; Table S3).

**Figure S3:**
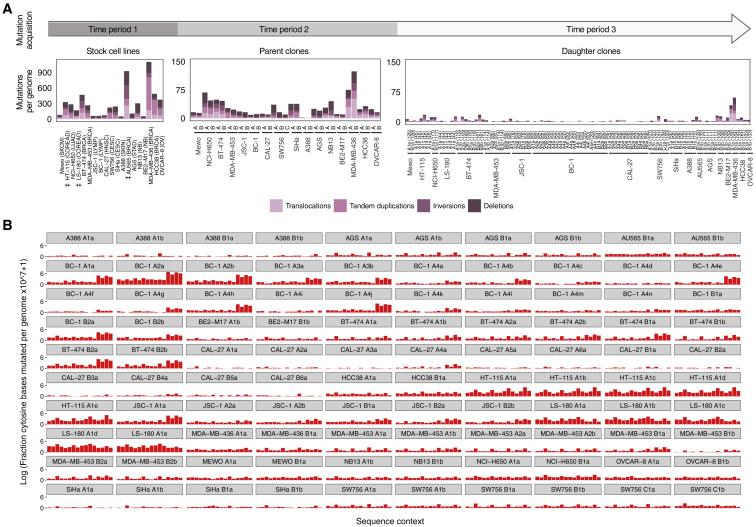
Rearrangements and C>T Substitutions at NCG Contexts, Associated with SBS1 and 5-Methylcytosine Deamination, Are Generated over Time, Related to Figure 3 Examination of additional mutation types acquired over time in cell line samples subjected to whole-genome sequencing and experimental design in Figure 2. (A) Bars indicate the numbers of color-coded rearrangement classes acquired during the time periods outlined in Figure 2, in indicated cell lines. Daughter clones were cultivated for the number of days indicated in brackets. ‡ Only single parent clones from HT-115, LS-180 and AU565 cell lines were subject to whole-genome sequencing and their sequences were used as proxies for the mutational catalogs of the corresponding stock cell lines. (B) Each panel displays the fraction of the cytosine (or guanine) bases at 16 possible trinucleotide contexts that were mutated to thymines (or adenines, respectively) over the examined *in vitro* periods (*Period 3*; Figure 2), in 100 indicated daughter and granddaughter clones.

SBS3 is a relatively flat and featureless base substitution signature (Figure S1) that is associated with defective homologous recombination-based DNA repair and inactivating mutations of *BRCA1* and *BRCA2* (Alexandrov et al., 2013a, Nik-Zainal et al., 2012a). It is usually accompanied by small deletions with overlapping microhomology at their boundaries and large numbers of rearrangements, including tandem duplications and deletions (Nik-Zainal et al., 2012a, Nik-Zainal et al., 2016). The breast cancer cell line MDA-MB-436 is deficient in BRCA1 (Elstrodt et al., 2006) and generated SBS3 during *in vitro* culture accompanied by the characteristic deletions with microhomology and large numbers of rearrangements (Figures 3 and S3A). SBS3 was also generated in the ovarian (OVCAR-8) and breast (HCC38) cancer cell lines (Figure 3), which have attenuated *BRCA1* expression due to promoter methylation as well as in lung adenocarcinoma (NCI-H650) and breast cancer (AU565) cell lines, which did not show obvious deficiencies in BRCA1 or BRCA2 function (Figure S2; Table S4). In contrast to MDA-MB-436, however, SBS3 in these lines was not accompanied by substantial numbers of deletions with microhomology or rearrangements (Figure S3B).

SBS1 and SBS5 have previously been attributed to processes generating mutations throughout life in normal tissues at constant rates in all individuals (Alexandrov et al., 2015). SBS5, which is of unknown origin, was identified in most clones (Figures 3B and 3C). SBS1, which is attributed to deamination of 5-methyl cytosine (Alexandrov et al., 2013a, Pfeifer, 2006), was not detected by computational analysis among *in vitro*-generated mutations. However, the distinctive profile of SBS1, characterized by C>T mutations at NCG trinucleotides (mutated bases underlined and referred to by the pyrimidine partner of the mutated base pair; N any base) was clearly visible following normalization of mutation frequencies to account for depletion of NCG trinucleotides in the human genome (Figure S3B; STAR Methods), indicating that the underlying process continues to operate in all cell lines but is not detected because of the relatively small numbers of mutations generated.

SBS18 is prominent in neuroblastoma (Alexandrov et al., 2013a) and continued to be generated in all of the neuroblastoma cell lines examined (Figure 3). It was also, however, observed in many daughter clones that were whole-genome sequenced (and thus captured sufficient numbers of mutations) of cell lines in which it was not detected in stocks (Figure 3). It therefore appears to be a common feature of *in vitro* culture, as previously noted (Rouhani et al., 2016). SBS18 may be generated by DNA damage caused by reactive oxygen species (Viel et al., 2017), and this mechanism could plausibly mediate its manifestation as a consequence of *in vitro* cell culture.

#### Activities of APOBEC-Associated Signatures 2 and 13

SBS2 and SBS13 have been attributed to APOBEC cytidine deaminase activity and are common in human cancer, although the factors responsible for their activation remain mysterious (Alexandrov et al., 2013a, Nik-Zainal et al., 2012a, Roberts et al., 2013). SBS2 and SBS13 show substitutions of cytosine at TCN trinucleotides, with SBS2 predominantly characterized by C>T and SBS13 by C>G and C>A mutations. The C>T transitions may arise by replication of uracils generated by APOBEC cytidine deamination, whereas the C>G, C>A, and potentially additional C>T substitutions may be introduced by error-prone polymerases following uracil excision and generation of abasic sites by uracil-DNA glycosylase (UNG) (Helleday et al., 2014, Roberts and Gordenin, 2014).

The rates of acquisition of SBS2 and SBS13 during *in vitro* culture were highly variable between cancer cell lines, between different daughter lineages of the same cancer cell line, and over time in the same daughter lineages (Figure 3; Table S3). Despite the clear presence of these signatures in the stocks of the cervical (SW756 and SiHa) and the breast (AU565) cancer cell lines, there was no discernible continuing activity in their daughter lineages. Of the remaining eight cell lines with prominent APOBEC activity in stocks, continuing generation of SBS2 and SBS13 was detectable in three breast cancers (BT-474, MDA-MB-453, HCC38), two B cell lymphomas (BC-1, JSC-1), a squamous carcinoma of the tongue (CAL-27), a lung adenocarcinoma (NCI-H650), and possibly a cutaneous squamous cell carcinoma (A388).

**Figure 4:**
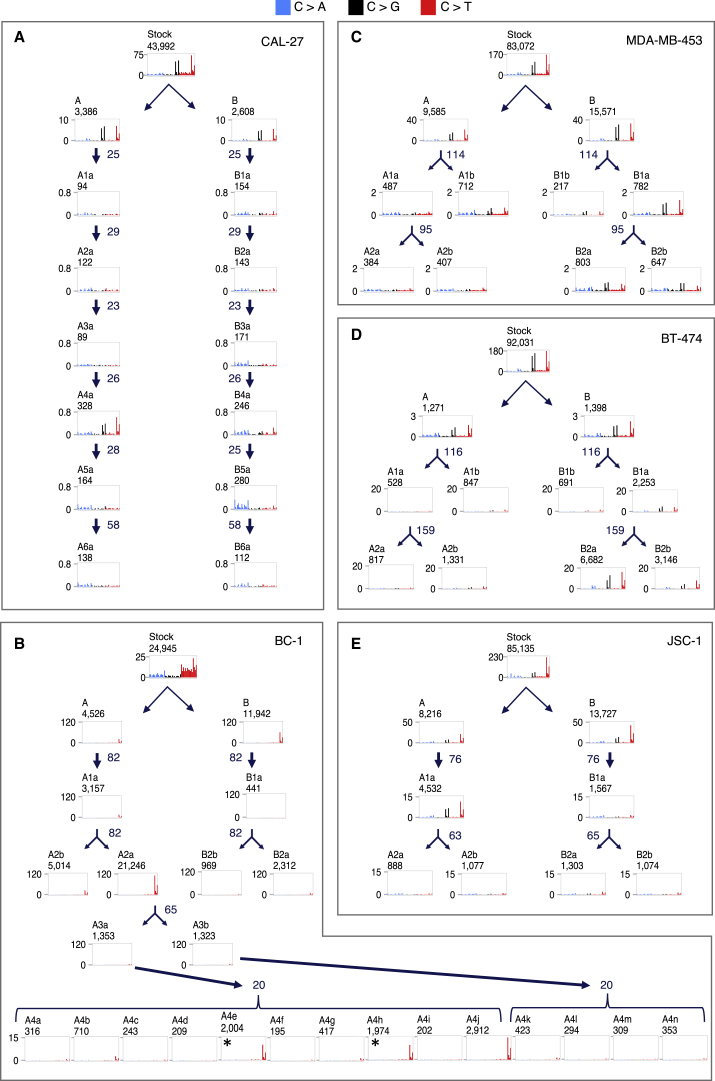
Serial Cloning Reveals the Episodic Nature of APOBEC Mutagenesis (A–E) Patterns of base substitutions acquired in five cancer cell lines (A, CAL-27; B, BC-1; C, MDA-MB-453; D, BT-474; E, JSC-1) during the serially examined consecutive time frames (see Figure 2). Numbers of days between individual single-cell cloning events are indicated in blue and represent the time periods allowed for an *in vitro* acquisition of mutations captured in mutational catalogs of daughter or granddaughter clones (*period 3*, Figure 2). Mutational catalogs display only mutations at cytosine bases, and their total number is indicated at the top of each panel. x axes indicate the sequence contexts immediately 5′ and 3′ to the mutated cytosine base in the alphabetical order (ACA, ACC, ACG, ACT, CCA, CCC, CCG, CCT, GCA, GCC, GCG, GCT, TCA, TCC, TCG, TCT). y axes indicate the counts of mutations acquired genome-wide (×10^−3^). See Figure 3 and Table S3 for annotation of mutational signatures in all samples. Indicated clones (^∗^) share the majority of mutations.

To investigate further this variability of APOBEC-associated signature activity, we subjected two breast cancer (MDA-MB-453, BT-474) and two B cell lymphoma (BC-1, JSC-1) cell lines to further rounds of subcloning and the squamous carcinoma of the tongue cell line (CAL-27) to seven serial rounds of subcloning over short periods of time (Figure 4; signatures annotation in Figure 3 and Table S3). These experiments confirmed that substantial differences in numbers of APOBEC-associated mutations often occur between daughter clones from the same parent. For example, the burden of APOBEC-associated signatures acquired across multiple clones (A4a–A4j) derived from a single clone (A3a) from the BC-1 cell line, following 20 days of *in vitro* culture, varied >100-fold (Figure 4B; Table S3).

**Figure S4:**
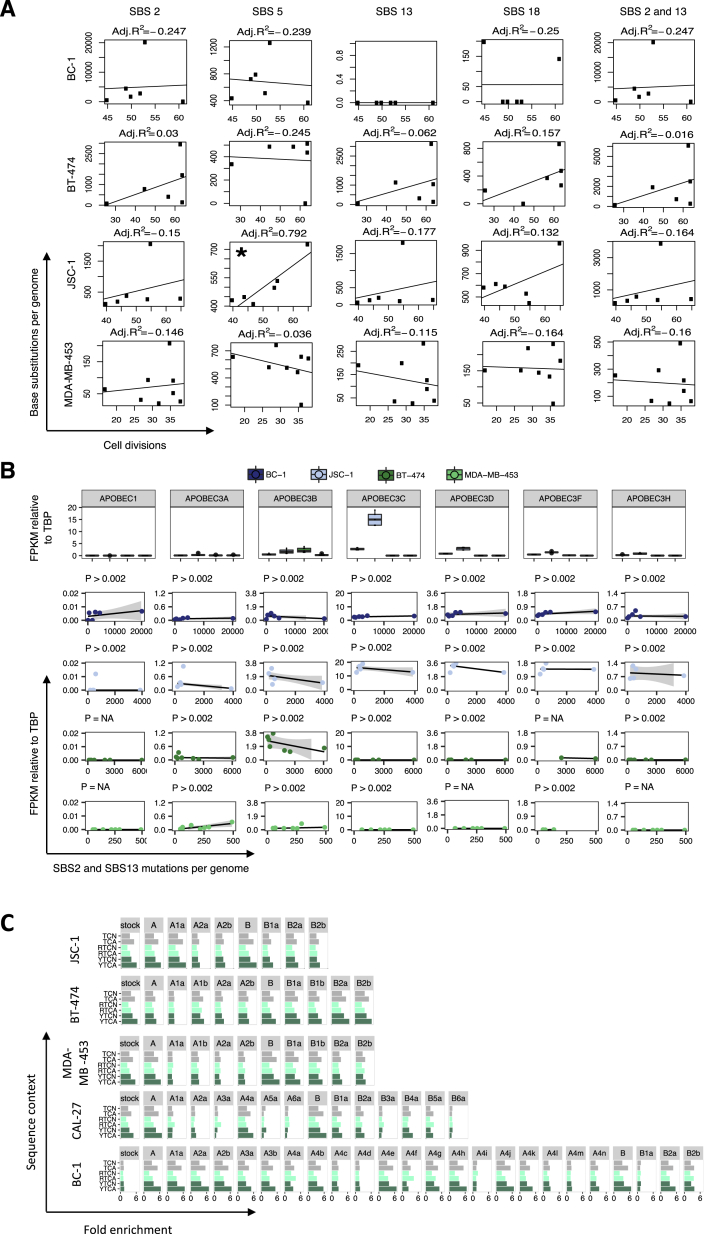
Episodic APOBEC Mutagenesis Is Likely Mediated by APOBEC3A, but It Does Not Depend on Proliferation Rates or Expression of APOBEC Genes in Examined Cell Lines, Related to Figures 3 and 4 (A) Cell divisions were measured for 26 daughter and granddaughter clones from the indicated cell lines and compared to the genome-wide burdens of the indicated signatures acquired during the examined *in vitro* time frames (*Period 3*, Figure 2). The best fit, as well the adjusted R^2^, are indicated in plots where sufficient data points were generated for a statistical comparison. ^∗^p < 0.05. (B) RNA-sequencing derived transcription levels (FPKM = Fragments Per Kilobase of transcript per Million mapped reads) of APOBEC family members with documented deaminase activity on DNA and preference to induce mutations at TCN context were examined in clones from color-coded cell lines, where RNA-sequencing data was generated (Table S2). Only those clones were considered where sufficient data was generated to accurately derive point estimate expressions of examined genes (STAR Methods). Expression was standardized relative to TATA-binding protein (*TBP*). Top panel: expression of *APOBEC* genes in clones from four indicated cell lines. Horizontal bars indicate the median expression level. Bottom panels: Expression of *APOBEC* genes was compared to the total burden of SBS2 and SBS13 mutations acquired genome-wide *in vitro*, in daughter and granddaughter clones from indicated cell lines. Robust regression was applied to derive the best estimates for the slopes of the indicated signatures (black lines), 95% confidence intervals (gray shading) and indicated *P* values, all of which were above the Bonferroni threshold corresponding to significance at the 0.05 level, p = 0.002 (corresponding to 0.05/23, where 23 is the number of successful tests). In some cases, insufficient data points were generated for a statistical comparison (p = NA). (C) Each panel represents enrichment of genome-wide C>T and C>G mutations in indicated clones, at SBS2 and SBS13-specific sequence contexts (TCN, TCA) and at motifs associated with APOBEC3A or APOBEC3B-indeced mutagenesis (YTCN/YTCA and RTCN/RTCA, respectively). N is any base, R is any purine and Y any pyrimidine base. A and B are parent clones, others are daughter and granddaughter clones from the related lineages.

Fluctuation in numbers of APOBEC-associated mutations was also observed between different phases of individual cell lineages. For example, daughter A1a from the BC-1 cell line acquired 3,157 mutations at cytosine bases in 82 days, the majority of which were SBS2 and SBS13 mutations (Figure 4B). During its succeeding round of propagation, which was for the same period of time, one of the two granddaughter clones acquired 21,246 of such mutations and the other 5,014. Indeed, in the daughter lineages of some cell lines, mutational activity appeared to cease completely before reactivating and then discontinuing again (although we cannot exclude the presence of extremely small numbers of mutations). This intermittent temporal pattern of activity was most obvious during the serial propagation of CAL-27, in which evidence of APOBEC mutagenesis was clearly seen during one period of lineage A (clone A4a) and was absent from at least three others (Figure 4A). The variation in activities of SBS2 and SBS13 was not obviously associated with differences in cell-proliferation rates (Figure S4A) and was in marked contrast to other signatures acquired *in vitro* in the same set of clones (Figure 3; Table S3). The results indicate that SBS2 and SBS13 mutations can be generated in short, intense bursts of activity with long intervening periods of silence, a pattern that we have termed “episodic mutagenesis.”

Most cancer cell lines with APOBEC activity displayed C>T, C>G, and C>A mutations at the characteristic sequence contexts (Figure 4), and thus a combination of SBS2 and SBS13, as do most primary human cancers in which APOBEC mutagenesis is found (Alexandrov et al., 2013a). However, the B cell lymphoma cell line BC-1 and its daughter cell lineages exhibited exclusively C>T mutations (Figure 4B). Analysis of methylation and expression data demonstrated that *UNG* is expressed at extremely low levels in BC-1 due to promoter methylation (Figure S2; Table S4; STAR Methods). These data indicate that UNG activity is required for the generation of C>G and C>A mutations following cytosine deamination in human cells, consistent with previous reports from engineered model systems (Chan et al., 2012, Di Noia and Neuberger, 2007, Taylor et al., 2013), and thus add further weight of evidence to the hypothesis that APOBEC deaminases are the sources of SBS2 and SBS13 in human cancer.

### *Kataegis* Is Generated during *In Vitro* Culture

**Figure 5:**
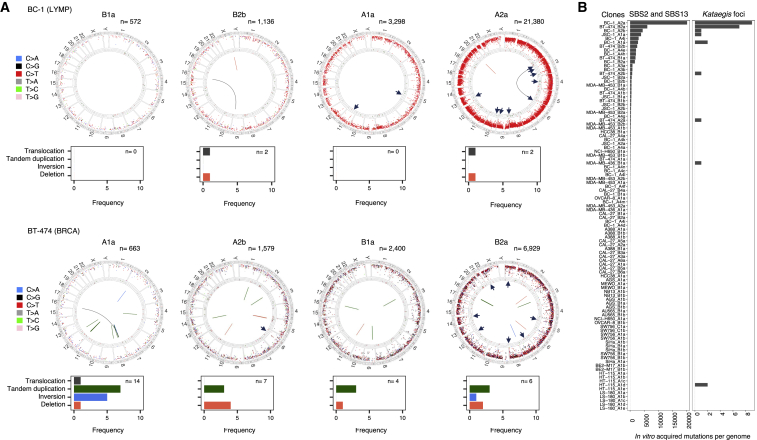
Genome-wide and Localized Foci of APOBEC-Associated Mutations, *Kataegis*, Are Generated *In Vitro* (A) Circos plots depict mutations acquired *in vitro* in exemplar daughter or granddaughter clones. Color-coded base substitutions are plotted as dots in rainfall plots (log intermutation distance), and their total numbers are indicated. Short green lines, short insertions; short red lines, short deletions. Arrows point to examples of *kataegis*. Central lines indicate rearrangements, color coded and quantified in the bar charts at the bottom. BRCA, breast carcinoma; LYMP, lymphoma B cell. (B) Bars display frequencies of *in vitro*-acquired *kataegis* foci and the total burden of genome-wide APOBEC-associated signatures (SBS2 and SBS13) across 100 whole-genome-sequenced daughter and granddaughter clones from indicated cell lines. For durations of the examined *in vitro* time frames related to the samples displayed in (A) and (B), see Table S2.

Most mutations caused by APOBEC mutagenesis during *in vitro* culture were approximately evenly distributed over the genome, recapitulating the pattern generally observed in cancers *in vivo* (Figure 5A) (Alexandrov et al., 2018). Foci of localized APOBEC-associated hypermutation (Nik-Zainal et al., 2012a, Roberts et al., 2012), *kataegis*, were also acquired during *in vitro* culture of some cell lines (Figure 5) predominantly occurring in clones with genome-wide SBS2 and SBS13 and with more foci in samples with higher rates of genome-wide mutagenesis (Figure 5). The locations of *kataegis* foci in cancer genomes have previously been associated with the presence of rearrangements (Nik-Zainal et al., 2012a, Roberts et al., 2012), and many acquired during *in vitro* culture also clearly co-localized with genome rearrangements (Figure 5A). Multiple *kataegis* foci composed exclusively of C>T mutations were acquired in the UNG-deficient BC-1 cell line (Figure 5A), indicating that formation of UNG-mediated abasic sites is not required for their genesis.

#### Origins of APOBEC-Associated Mutational Signatures 2 and 13

The particular APOBEC family member(s) that causes the SBS2 and SBS13 mutations observed in cancer is currently a matter of discussion. *APOBEC3B* has been implicated because its expression levels correlate most strongly with the burden of putative APOBEC-induced mutations (Burns et al., 2013a, Burns et al., 2013b, Roberts et al., 2013). However, a germline polymorphism that effectively deletes *APOBEC3B* and stabilizes expression of *APOBEC3A* has been associated with higher burdens of SBS2 and SBS13 in breast cancers and indicates that, at least in some cancers, APOBEC3B cannot be responsible (Caval et al., 2014, Nik-Zainal et al., 2014). Indeed, the extended sequence context in which SBS2 and SBS13 mutations occur in human cancers is predominantly that at which APOBEC3A, rather than APOBEC3B, induces mutations in yeast (TCN preceded by a pyrimidine base rather than a purine) (Chan et al., 2015). The SBS2 and SBS13 mutations generated in the cancer cell lines *in vitro* were largely enriched in the YTCN/YTCA sequence context (where Y is a pyrimidine), consistent with episodic mutagenesis mainly being mediated by APOBEC3A (Figure S4C; STAR Methods).

To explore the possibility that changes in APOBEC gene expression mediate the observed fluctuation in APOBEC mutation rates, we conducted RNA sequencing of daughter and granddaughter clones from two breast cancer (MDA-MB-453, BT-474) and two B cell lymphoma cell lines (BC-1, JSC-1) (STAR Methods). The clear correlations between the burdens of *in vitro*-acquired SBS2 and SBS13 mutations and expression levels of *APOBEC* genes were not detected (Figure S4B). It remains possible, however, that transient changes in mRNA levels had occurred that were not captured by these measurements. The episodic pattern of APOBEC mutagenesis, and hence the potentially fluctuating nature of underlying APOBEC expression, may explain the absence of detectable *APOBEC3A* expression in the daughter clones showing ongoing generation of SBS2 and SBS13, despite evidence for APOBEC3A being responsible from the sequence context of the mutations. Indeed, it may also explain the lack of common upregulation of *APOBEC3A* expression in human cancers and its weak correlation with SBS2 and SBS13 mutation burdens *in vivo* (Burns et al., 2013a, Roberts et al., 2013).

APOBEC3 cytidine deaminases have been implicated in innate immunity and in restricting retrotransposons and viruses through DNA-editing-dependent and independent mechanisms (Vieira and Soares, 2013, Conticello, 2008). It is conceivable that the SBS2 and SBS13 mutations observed in cancers *in vivo* and during *in vitro* culture represent collateral mutational damage on the host cell genomes from APOBEC-mediated responses directed against viruses and/or retrotransposons. The BC-1 and JSC-1 B cell lymphoma lines, which continue to generate SBS2 and SBS13 in culture, both carry Epstein-Barr virus (EBV) and human herpesvirus-8 DNA sequences (STAR Methods) and express viral transcripts (Callahan et al., 1999, Cannon et al., 2000, Cesarman et al., 1995). Truncated versions of HPV16 and HPV18 were found in the cervical cancer cell lines SiHa and SW756 (STAR Methods), respectively and as reported previously (Schneider-Gädicke and Schwarz, 1986, Yee et al., 1985), with no detectable ongoing APOBEC-associated mutagenesis. However, viral sequences were not detected in the breast (MDA-MB-453 and BT-474) and squamous carcinoma of the tongue (CAL-27) cell lines, which continued to acquire APOBEC-associated signatures in culture (STAR Methods). Thus, among the small number of cell lines studied here, the presence of examined viruses is not required to trigger APOBEC mutagenesis. The continued acquisition of SBS2 and SBS13 during *in vitro* cell culture also indicates that microenvironmental influences, such as inflammation, are unlikely to be necessary for APOBEC-mediated mutagenesis, although we cannot exclude the possibility that they play a role *in vivo* (Vieira and Soares, 2013).

**Figure S5:**
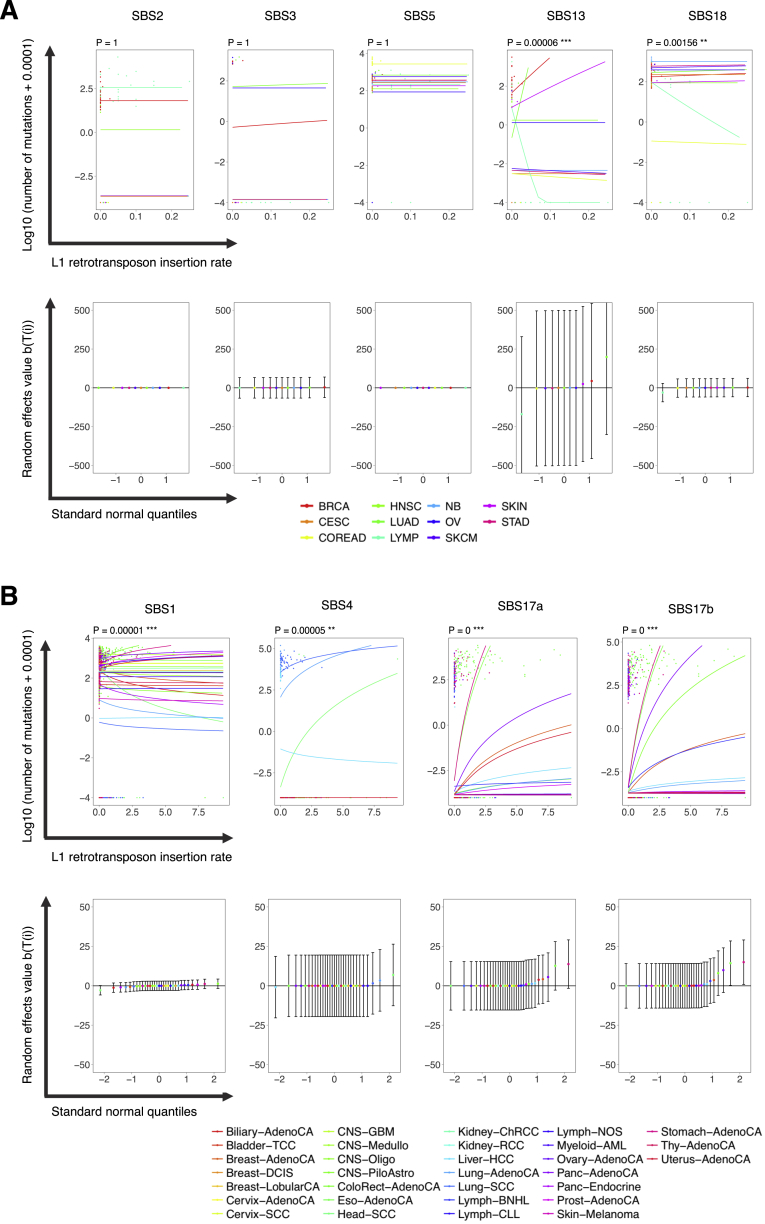
Significant Relationships between Somatic Retrotransposition and Mutational Signatures in Cell Lines and Primary Cancers, Related to Figures 3 and 4 (A and B) The upper plots in both panels show the dependence of the observed numbers of mutations assigned to the indicated signatures (dots), and fitted values (lines) estimated using the GLMM Poisson regression model (STAR Methods), on the L1 insertion rate in cell line clones (panel A) and primary cancer samples (panel B). P values which fall below the Bonferroni thresholds corresponding to significance at the 0.05, 0.01, and 0.001 levels are indicated as ^∗^, ^∗∗^ and ^∗∗∗^, respectively. The bottom plots show the estimated effects of cell line (panel A) or primary cancer (panel B) types on the slope of the regression line, in ranked order, against the normal quantiles. For each tumor type, the fitted value is accompanied by a 95% confidence interval. See Table S5 for cell line and primary cancer samples considered in analyses.

We further quantified *in vitro*-acquired insertions of the long interspersed nuclear elements LINE−1 (L1) retrotransposons using the whole-genome sequence data from available clones and examined the relationship between retrotransposition mobilization and APOBEC-associated signatures (STAR Methods; Table S5). This analysis revealed a significant correlation between the rates of *in vitro*-acquired L1 insertions and burdens of SBS13 and SBS18 (respectively, p < 0.001/5 and p < 0.01/5; Bonferroni-adjusted), most pronounced in breast and lung adenocarcinoma cell lines (Figure S5; Table S5A). To further investigate this observation, we extended the analysis to 2,353 primary cancers from most cancer types where somatic retrotransposon insertions and mutational signatures have been annotated previously as part of the International Cancer Genome Consortium Pan-Cancer Analysis of Whole Genomes (ICGC PCAWG; STAR Methods) (Alexandrov et al., 2018, Rodriguez-Martin et al., 2017). There were significant correlations between the rates of somatic retrotransposition and individually SBS1, SBS4, SBS17a, and SBS17b (p < 0.01/31 for SBS4 and p < 0.001/31 for SBS1, SBS17a, and SBS17b; Bonferroni-adjusted, Figure S5B; Table S5). However, there was no evidence of a correlation for SBS13 (p = 1.0). The cell line data suggest a possible relationship between APOBEC mutagenesis and retrotransposition activities, although more direct experimental testing is required to establish this. Variable strengths of the effects observed across the analyzed cell line types (Figure S5) suggest that other factors are involved. Indeed, retrotransposition is common in colorectal cancers, where APOBEC mutagenesis is rare (Alexandrov et al., 2013a, Tubio et al., 2014).

### The Prevalence of Continuing APOBEC Mutagenesis

To explore further the observation that continuing APOBEC mutagenesis was not observed in at least three cell lines in which it represented the predominant mutational process in the past (SiHa, SW756, and AU565; Figure 3; Table S3), we used single-cell sequencing as an alternative approach to single-cell cloning, which is laborious and unpredictable, with some cell lines being amenable to cloning and others not.

Single cells, equivalent to parent clones, after subtraction of mutations present in the stock cell line, provide information on mutations acquired since its most recent clonal expansion (Figure 2; STAR Methods). However, the small amounts of DNA obtained from single cells necessitate whole-genome amplification (WGA) before sequencing, and current amplification technologies entail both non-amplification of a substantial proportion of the genome and introduction of artifact mutations (Gawad et al., 2016). Nevertheless, mutational signature analysis does not require detection of all mutations in a genome. Moreover, the impact of artifacts can be mitigated if the signature of interest is known *a priori*, has a distinctive profile (as for SBS2 and SBS13), and can be deconvoluted from signatures of genome amplification-induced mutations.

**Figure 6:**
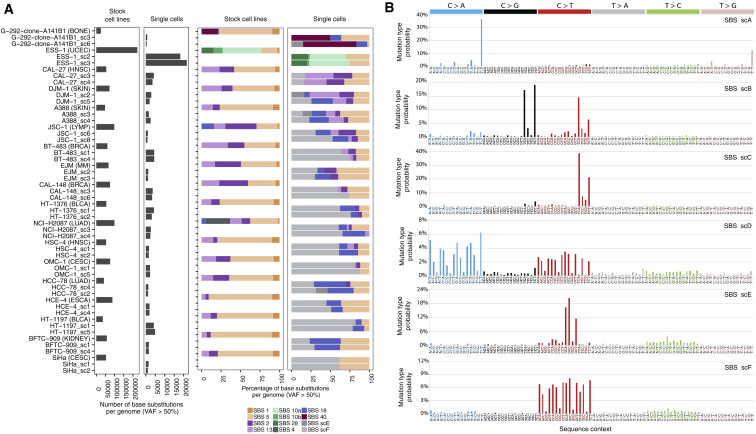
Mutational Signatures in Single Cells Indicate Commonly Continuing APOBEC-Associated Mutagenesis (A) Bars represent the numbers of base substitutions (left) and mutational signatures (right) in whole-genome-sequenced stock cell lines and their single cells (cancer type abbreviations in Table S2). Mutations presenting at <50% VAF (variant allele fraction) were excluded from mutational catalogs from single cells, as they mostly formed patterns of signatures scE and scF, likely introduced during the process of the single-cell DNA preparation (STAR Methods; see Figure S6A for mutational signatures annotation using complete catalogs). (B) Mutational signatures extracted from the complete mutational catalogs from 36 whole-genome-sequenced single cells. Each signature is displayed according to the 96-substitution classification on horizontal axis, defined by the six color-coded substitution types and sequence context immediately 5′ and 3′ to the mutated base. Vertical axes show the percentage of mutations attributed to specific mutation types.

Hence, to determine the proportion of cell lines that experienced APOBEC mutagenesis at some point following the establishment of the most recent common ancestor of the stock cell line, we whole-genome sequenced 32 single cells from 16 cancer cell lines exposed to it in the past, alongside single cells from endometrial cell line with POLE-associated SBS10a-b and SBS28 (ESS-1) and an osteosarcoma cell line (G-292-clone-A141B1) with SBS40, which is characterized by roughly uniform representation of all 96 mutation types (Figure 6A; STAR Methods).

**Figure S6:**
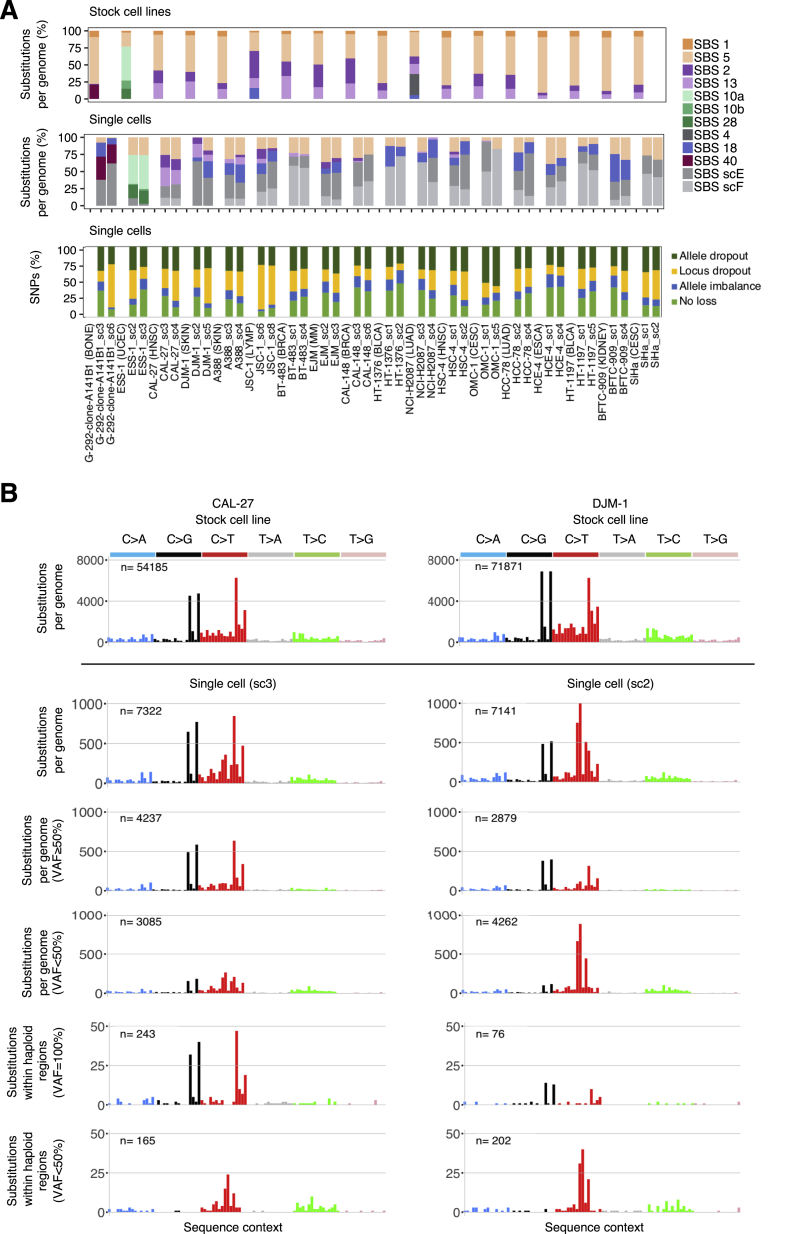
Signatures of False-Positive Somatic Mutations Are Present in DNA Prepared from Single Cells, Related to Figure 6 (A) Top two panels: bars represent the percentage of base substitutions attributed to color-coded signatures in complete (rather than filtered, see Figure 6A) mutational catalogs from whole-genome sequenced stock cell lines from the denoted cancer classes (abbreviations in Table S2) and their single cells. The bottom panel represents the color-coded fractions of minor alleles at examined heterozygous SNP loci, in indicated single cells, which were (i) lost due to WGA-associated locus dropouts, (ii) lost due to WGA-associated allele dropouts or (iii) fall under the detection threshold for identification of base substitutions due to WGA-associated imbalanced amplification. (B) Spectra of mutations identified genome-wide in two exemplar stock cell lines (top panels) and in their corresponding single cells (bottom panels), genome-wide or within haploid regions at the indicated variant allele fractions (VAF). Each panel is displayed according to the 96-substitution classification on the horizontal axis defined by the six color-coded substitution types and sequence context immediately 5′ and 3′ to the mutated base. Order of the sequence context follows the standard alphabetical representation (see Figure 6B). Total number of base substitutions is indicated on the top of each panel. C>T variants at NCG contexts and T>C mutations at ATN contexts in stock cell lines largely represent germline variation due to the non-availability for most cancer cell lines of normal DNAs from the same individuals.

Deconvolution of the single-cell mutational catalogs revealed six signatures (Figure 6B; STAR Methods), including those associated with mutations in *POLE* (scA) and APOBEC activity (scB and scC) and a signature (scD) characterized in part by C>A mutations similar to SBS18, which probably results from *in vitro* culture (see above). Two novel signatures (scE and scF) were present exclusively in single cells and absent from the corresponding stock cell lines (Figure S6). These likely represent variants introduced during the WGA process and cell lysis and were in large part removed for subsequent attribution of mutational signatures to individual cells (Figure 6A; Table S3; STAR Methods).

Attribution of mutational signatures revealed the *POLE-*associated SBS10a-b and SBS28, as well as SBS40 only in single cells from the ESS-1 and G-292-clone-A141B1 cell lines carrying these signatures, respectively (Figure 6A; Table S3). APOBEC-associated signatures were identified in single cells from cell lines shown in our previous analyses to have continuing activity (CAL-27 and JSC-1) but not in single cells from ESS-1 cell line in which APOBEC mutagenesis had never been present or in single cells from the cervical cell line SiHa in which mutagenesis had ceased (Figures 3 and 6A; Table S3). Furthermore, SBS4, related to tobacco smoking, was not detected in single cells from the lung adenocarcinoma cell line NCI-H2087 in which this signature was present in the stock sequence (Figure 6A; Table S3). Thus, mutational signatures detected in single cells indicate continuing mutagenesis. On the basis of detection in at least one single cell, we estimate that at least ∼75% of cancer cell lines that experienced APOBEC mutagenesis in the past (before the most recent common ancestor of the stock population, as revealed by the sequence of the stock cell line) have continued to generate SBS2 and SBS13 at some point following the establishment of their most recent common ancestor cells (as revealed by mutations present in the single cells, but not the stocks).

## Discussion

The cancer cell lines and PDX models analyzed here now provide a substantial resource for exploring mechanistic questions relating to the mutational processes underlying the majority of mutational signatures. Given the high prevalence of SBS2 and SBS13 in human cancer (Alexandrov et al., 2013a), the nature of the biological factors that instigate APOBEC mutagenesis is an important question. Cell lines that continue to generate these signatures over time represent models suitable for such explorations. For example, systematic knockouts of APOBEC family members in such cell lines offer an opportunity to unambiguously establish a causative link between APOBEC enzymes and mutagenesis in human cancer cells and to resolve the current discrepancies in the field concerning which member may be responsible (Burns et al., 2013a, Burns et al., 2013b, Chan et al., 2015, Middlebrooks et al., 2016, Nik-Zainal et al., 2014, Starrett et al., 2016). Our studies support the propositions that APOBEC-associated SBS2 and SBS13 can be generated at multiple time points during the evolution of individual cancers, as suggested previously by studies showing that they can be acquired both early and late but are sometimes restricted to one temporal phase of cancer development (often late) (Jamal-Hanjani et al., 2017, McGranahan et al., 2015, Nik-Zainal et al., 2012b).

We further find that APOBEC-associated mutagenesis exhibits a highly fluctuant mutation rate over time and can be episodic *in vitro*, operating in short bursts with long periods of inactivity. The extent to which such episodic mutagenesis occurs *in vivo* awaits further investigation. Approaches using multiregional sequencing or serial sampling of primary cancers may, however, incompletely report the episodic nature of the mutagenesis pattern, due to the inability to track mutation acquisition over relatively short periods of time. Nevertheless, the observed variability in SBS2 and SBS13 mutations between branches of phylogenetic trees from primary tumors (Jamal-Hanjani et al., 2017, Lee et al., 2017) lends support to the possibility that some or all APOBEC-associated mutagenesis *in vivo* is episodic.

The variability in acquisition of APOBEC-associated mutational signatures occurs in cell culture despite the absence of many proposed initiators and regulators of APOBEC activity, including the immune system and detectable exogenous virus infections (Kanu et al., 2016, Middlebrooks et al., 2016, Vieira and Soares, 2013), the close genetic relationships of the cell lineages examined, and a highly controlled and uniform tissue culture microenvironment shared by sister lineages with very different mutation rates, and is in marked contrast to other ongoing mutational signatures. It is unlikely that episodic APOBEC activity was induced simply by the single-cell cloning process because individual lineages were cloned multiple times and APOBEC-associated signatures were not always detected. Moreover, if mutational bursts occurred during the isolation of single parent cells, most mutations would be shared between daughters. The data therefore suggest that the initiators of APOBEC mutagenesis *in vitro* are cell intrinsic and have intermittent and irregular activity. It is possible that these may also operate *in vivo* and may include modulators of availability of single-stranded DNA (ssDNA) substrate, changing access of APOBEC(s) to nuclear DNA and retrotransposon mobilization.

## STAR★Methods

### Key Resources Table

**Table:** 

REAGENT or RESOURCE	SOURCE	IDENTIFIER
**Biological Samples**		

1,001 human cancer cell lines	COSMIC Cell Line Project	https://cancer.sanger.ac.uk/cell_lines

**Critical Commercial Assays**		

Custom SureSelect Library Prep Kit	Agilent	930075
SureSelect Targeted Enrichment kit	Agilent	5190-4394
illustra GenomiPhi V2 DNA amplification kit	GE Healthcare	45-001-222
Ribo-Zero rRNA Removal Kit	Illumina	RZH110424
KAPA Stranded mRNA-Seq Kit	Kapa Biosystems	07962193001
ERCC RNA Spike-In Mix	Thermo Fisher	4456740

**Deposited Data**		

DNA sequence data	This paper	EGA: EGAD00001004201, EGAD00001004203
RNA sequence data	This paper	EGA: EGAD00001004202

**Software and Algorithms**		

Mapping workflow	Cancer, Aging and Somatic Mutation group, Wellcome Sanger Institute	https://dockstore.org/containers/quay.io/wtsicgp/dockstore-cgpmap
Mutation calling workflows	Cancer, Aging and Somatic Mutation group, Wellcome Sanger Institute	Whole-exome: https://dockstore.org/containers/quay.io/wtsicgp/dockstore-cgpwxs Whole-genome: https://dockstore.org/containers/quay.io/wtsicgp/dockstore-cgpwgs
vafCorrect	Cancer, Aging and Somatic Mutation group, Wellcome Sanger Institute	https://github.com/cancerit/vafCorrect
GOTTCHA	Freitas et al., 2015	https://github.com/poeli/GOTTCHA
TraFiC-mem; V.1.1.0		https://gitlab.com/mobilegenomes/TraFiC
TopHat2; V.2.1.1	Kim et al., 2013	https://github.com/infphilo/tophat
Picard; V.1.60		https://broadinstitute.github.io/picard/
Cufflinks; V.1.0.2	Trapnell et al., 2010	https://github.com/cole-trapnell-lab/cufflinks

**Other**		

Mutational catalogs from 1,001 cancer cell lines; V.83	COSMIC Cell Line Project	https://cancer.sanger.ac.uk/cell_lines
Expression and methylation datasets from 1,001 cancer cell lines	Iorio et al., 2016	https://www.cancerrxgene.org/gdsc1000/
Mutational catalogs from 577 PDX models and 25 originating tumors; V.1.3	National Cancer Institute’s Patient-Derived Models Repository	https://pdmr.cancer.gov/default.htm
Mutational catalogs from 2,709 ICGC PCAWG primary cancers; Platinum version	This paper; catalogs shared by the Mutational Signatures group of the ICGC PCAWG Project (Campbell et al., 2017)	Table S3

### Contact for reagents and resource sharing

Further information and requests for resources and reagents should be directed and will be fulfilled by the Lead Contact, Michael R. Stratton (mrs@sanger.ac.uk).

### Experimental models and subject details

Table S2 lists primary cancer, cell line and PDX cancer datasets used, alongside their sources (where the data was downloaded) or sequencing/experimental information (where data were newly generated).

#### ICGC PCAWG Platinum dataset

Somatic mutational catalogs from 2,709 primary human cancers (Table S2) were generated as part of the Platinum version of the ICGC PCAWG project (Alexandrov et al., 2018) and were shared by the PCAWG Mutational Signatures Working Group (Table S3), alongside the patterns of mutational signatures (Table S1) extracted from this dataset and their annotation across individual samples (Table S3). The Platinum set of mutational signatures was annotated here on catalogs from all cancer cell line samples and PDX models. Annotations of mutational signatures across the ICGC PCAWG Platinum dataset were used alongside the previously identified somatic retrotransposition events across the same dataset (Rodriguez-Martin et al., 2017) to examine the relationships between burdens of APOBEC-associated SBS2 and SBS13 and mobilization of retrotransposons (Figure S5).

#### Publicly available cell line and PDX datasets

Mutational catalogs from 1,001 human cancer cell lines and 577 PDX models (and their 25 available originating tumors) listed in Table S2 were downloaded, respectively, from the COSMIC Cell Line Project (v.83; https://cancer.sanger.ac.uk/cell_lines) and the National Cancer Institute’s Patient-Derived Models Repository (NCI PDMR v1.3; https://pdmr.cancer.gov/default.htm) and used for annotation of mutational signatures (Figure 1; Table S3).

Expression and methylation datasets from 1,001 human cancer cell lines were generated as part of the COSMIC Cell Line Project and processed previously (Iorio et al., 2016). These datasets are available at https://www.cancerrxgene.org/gdsc1000/ and were anayzed here as per section ‘*1,001 cell line panel expression and methylation datasets’* to find putative aberrations in genes associated with mutational signatures in examined cell lines (Figure S2 and Table S4).

#### Cell line samples and sequence data generated in this study

Cancer cell lines used in this study originate from the cryopreserved aliquots of 1,001 cell lines, sourced previously from collaborators or public repositories and extensively characterized as part of the Genomics of Drug Sensitivity in Cancer (GDSC) (Garnett et al., 2012, Iorio et al., 2016) and COSMIC Cell Line (Forbes et al., 2017) projects. All cell lines were mycoplasma negative and fingerprinted by single nucleotide polymorphism (SNP) and short tandem repeat (STR) profiling (https://cancer.sanger.ac.uk/cell_lines).

A subset of 28 of these cell lines was used to establish 58 parent and 141 daughter clones, which were subject to whole-exome and/or whole-genome sequencing (Table S2), depending on the rates of the mutational processes examined, to study mutation acquisition over time (Figures 3–5). A different subset of 18 stock cell lines was subject to whole-genome sequencing, alongside their respective 36 single cells (Table S2), to examine the prevalence of frequently ongoing APOBEC mutagenesis (Figure 6). Finally, we performed RNA sequencing on 26 cell line clones (Table S2), to examine relationships between expression of APOBEC genes and burdens of the corresponding mutational signatures (Figure S4B).

### Methods Details

#### Cell culture and isolation of single cells

Cell were maintained at 37C°, 5% CO_2_ in RPMI or DMEM/F12 media (Iorio et al., 2016), supplemented with 5% fetal bovine serum and penicillin/streptomycin (GIBCO). Prior to cell sorting, cell lines were stained with Calcein AM (Cambridge Bioscience) and propidium iodide (PI) (Thermo Fisher) and Calcein-positive and PI-negative viable single cell isolated. Cells isolated for clonal expansions were sorted into media conditioned by 60%–80% confluent culture for 24 hours and filtered with low protein binding filters (Corning). Cells isolated for single cell sequencing were sorted into lysis buffer, as per manufacturer’s protocol (Illustra GenomiPhi V2; GE Healthcare).

#### DNA extraction and amplification

DNA was extracted using the DNeasy Blood and Tissue Kit (QIAGEN). Single cell DNA was amplified by multiple displacement amplification (MDA) as per the manufacturer’s protocol (Illustra GenomiPhi V2; GE Healthcare), using a two-hour amplification time. A no-cell negative control wells were processed with the same reagents to control for background contamination. Amplified DNA was purified using a 1:1 volume ratio of AMPure XP beads (Beckman Coulter) and DNA, following the manufacturer’s protocol.

#### Library preparation, sequencing and alignment

Genomic libraries were prepared with the Custom SureSelect Library Prep Kit (Agilent Technologies), aiming for an average insert size of approximately 500bp and 150bp for, respectively, whole-genome and whole-exome sequencing. The Sure Select Targeted Enrichment kit (Agilent Technologies) was used for exome enrichment. Following cluster generation, 150 base (whole-genome) and 75 base (whole-exome) paired-end sequence data was generated on Illumina HiSeq 2000/2500 (whole-exome) and HiSeq X Ten (whole-genome) platforms. Sequencing reads were aligned to the reference human genome (GRCh37) using the Burrows-Wheeler Alignment (BWA) tool (Li and Durbin, 2010), with ‘bwa mem’ settings. Unmapped, non-uniquely mapped reads and PCR-derived duplicate reads were excluded from further analysis. Small subset of samples was subject to random downsampling of the obtained sequence coverage for consistency between related samples. Table S2 provides the average sequence coverage considered for all samples.

#### Genotyping of cell line samples

All cancer cell lines were genotyped previously by SNP and STR profiling, as part of the COSMIC Cell Line Project (https://cancer.sanger.ac.uk/cell_lines). Individual clones obtained here were genotyped by comparing alleles presenting at the same set of SNP loci (not shown). Reads reporting alleles presenting at the enquired loci were quantified and pairwise comparisons generated among individual samples to confirm that all single-cell derived originate from their respective cell lines.

#### Mutation discovery

Base substitutions, indels and rearrangements were discovered using, respectively, the CaVEMan (Jones et al., 2016), cgpPindel (Raine et al., 2015) and BRASS algorithms developed in house (https://github.com/cancerit), by performing comparisons outlined in Table S2. Technology-specific artifacts and germline variants were removed by filtering against a panel of more than 100 unrelated normal samples available in-house. Additional post-processing filters were applied to remaining mutation calls as described previously (Nik-Zainal et al., 2012a). For X Ten-generated / BWA-mem aligned data, base-substitutions presenting at the loci where the median alignment score of mutation-reporting reads (ASMD) was < 130 were removed.

Numbers of mutant and wild-type reads presenting at the loci of the remaining base substitutions and indels were assessed across the related cell line samples using vafCorrect (Yates et al., 2017) and filters described below were subsequently applied to build catalogs of mutations acquired during the time frames of interest (Figure 2).

#### Mutational catalogs from stock cell lines and cell line clones

##### Mutational catalogs from daughter and descendant clones

Mutations were discovered in daughter clones by using their related parent clones as the reference sequences. Mutational catalogs from daughter clones therefore predominantly capture mutations acquired during the *in vitro* parts of the cellular lineages that are of known durations and span two single cell isolation steps used in establishment of the parent and daughter clones (see Figure 2 for further clarification). Smaller proportion of subclonal variants, acquired following expansion of the single daughter cells, may be captured too (see below, ‘*Clonality of the cell line clones’*). For some cell lines, further series of clonal generations were established to study mutations acquired during multiple consecutive *in vitro* time frames (Figures 3 and 4). Mutations in the subsequent generations of clones (for example, granddaughter clones) were discovered following the same principle of using the progenitor clones as reference sequences to reveal the mutations acquired during the *in vitro* time frames spanning the single cell isolation steps. Table S2 outlines all of the performed comparisons and durations of the examined *in vitro* time frames.

To retain mutations acquired predominantly *in vitro* and remove possibly pre-existing mutations acquired prior to this time frame, base substitutions and indels were considered only if (1) the mutation locus was covered in the reference clone by ≥ 15 reads, (2) mutation-reporting reads were not detected in the reference clone at variant allele fraction (VAF) ≥ 5%, (3) mutation-reporting reads were not detected in other available clones from the preceding part of the lineage at average VAF ≥ 5%, (4) mutation-reporting reads did not present at average VAF ≥5% across clones from the other available parent lineage(s) where mutation-reporting reads were detected, (5) mutation-reporting reads were not detected in sister clones derived from the same parent at average VAF ≥ 5% if the reference locus was covered by < 30 reads (whole-exomes only; in whole-genome experiments this step was omitted for consistency because sister clones were not always derived). Rearrangements were considered only if not detected in other related clones within 50bp of identified breakpoints.

##### Mutational catalogs from stock cell lines and parent clones – whole-exome sequencing

Mutational catalogs of both coding and non-coding variants from whole-exome sequenced cancer cell lines were downloaded from the COSMIC Cell Line Project (v.83; https://cancer.sanger.ac.uk/cell_lines). These were generated previously using an unrelated normal human genome as a reference sequence and were filtered extensively against the common germline variation (Iorio et al., 2016) (Figures 1 and 3). Mutational catalogs from stock cell lines therefore predominantly capture somatic mutations acquired within the lineages from the fertilized eggs to the most recent clonal expansions of individual stock cell lines and a proportion of germline variants due to the absence of the matched normal DNAs.

Mutations in whole-exome sequenced parent clones (Figure 3) were discovered by using their related stock cell lines as the reference sequences (comparisons in Table S2). The raw sequence data from the stock cell lines was available in-house from the COSMIC Cell Line project and realigned here for consistency with clones sequenced as part of this study to GRCh37 reference human genome, using Burrows-Wheeler Alignment (BWA) tool (Li and Durbin, 2010) with ‘bwa mem’ settings. Mutational catalogs from parent clones therefore predominantly capture mutations acquired during the time frames of unknown durations, which span establishment the most recent common ancestor cell of the stock cell line and isolation of the single parent cells (see Figure 2 for further clarification). Smaller proportion of subclonal variants, acquired following expansion of the single parent cells may be captured too (see ‘*Clonality of the cell line clones’*). To remove possibly pre-existing mutations acquired prior to this time frame, base substitutions and indels were only considered if (1) the mutated locus was covered in the corresponding stock cell line by ≥ 15 reads, (2) mutation-reporting reads were not detected in clones from the other available parent lineage(s) at average VAF ≥ 5%, (3) mutation-reporting reads were not detected in the corresponding stock cell line at VAF ≥ 5%.

##### Mutational catalogs from stock cell lines and parent clones – whole-genome sequencing

A subset of stock cell lines and subset of parent clones (from different sets of cell lines) were subject to whole-genome sequencing to be used as, respectively, references for their related single cells (Figure 6) and daughter clones (Figures 3 and 4). In the absence of the related reference sequence, mutations in whole-genome sequenced stock cell lines and parent clones were discovered using an unrelated normal human genome as a reference (see Table S2) and filtered against common germline variation listed in dbSNP (Sherry et al., 2001), 1000 genomes project (1000 Genomes Project Consortium et al., 2012), NHLBI GO Exome Sequencing Project (Fu et al., 2013) and 69 Complete Genomics panel (http://www.completegenomics.com/public-data/69-Genomes/) (Drmanac et al., 2010), as described previously (Alexandrov et al., 2013a), as well as against panels of additional 500 normal exomes and more than 120 normal genomes, by removing any mutations presenting in at least three well-mapped reads of at least two normal samples.

To derive proxies for mutational catalogs of the stock cell lines from which whole-genome sequenced parent clones were derived (Figures 3 and 4), we used mutations shared between the related parent clones, which pre-existed in their most recent common ancestor cell. Variants private to individual parent clones served as proxies for the lineages spanning establishment of the most recent common ancestor cell of the stock cell line and isolation of the single parent cells. Where only single parent clones were available (LS-180, HT-115, AU565 cell lines), they were used as mutational catalogs of the stock cell lines. Base substitutions and indels were classified as ‘private’, unless they were (1) identified by the variant calling algorithms in other parent clone(s) from the same cell line and (2) detected in > 2 samples from the other parent lineage(s) at VAF ≥ 5%. Rearrangements were classified as ‘private’, unless they were identified by BRASS in any of the clones derived from the other parent lineage(s).

**Figure S7:**
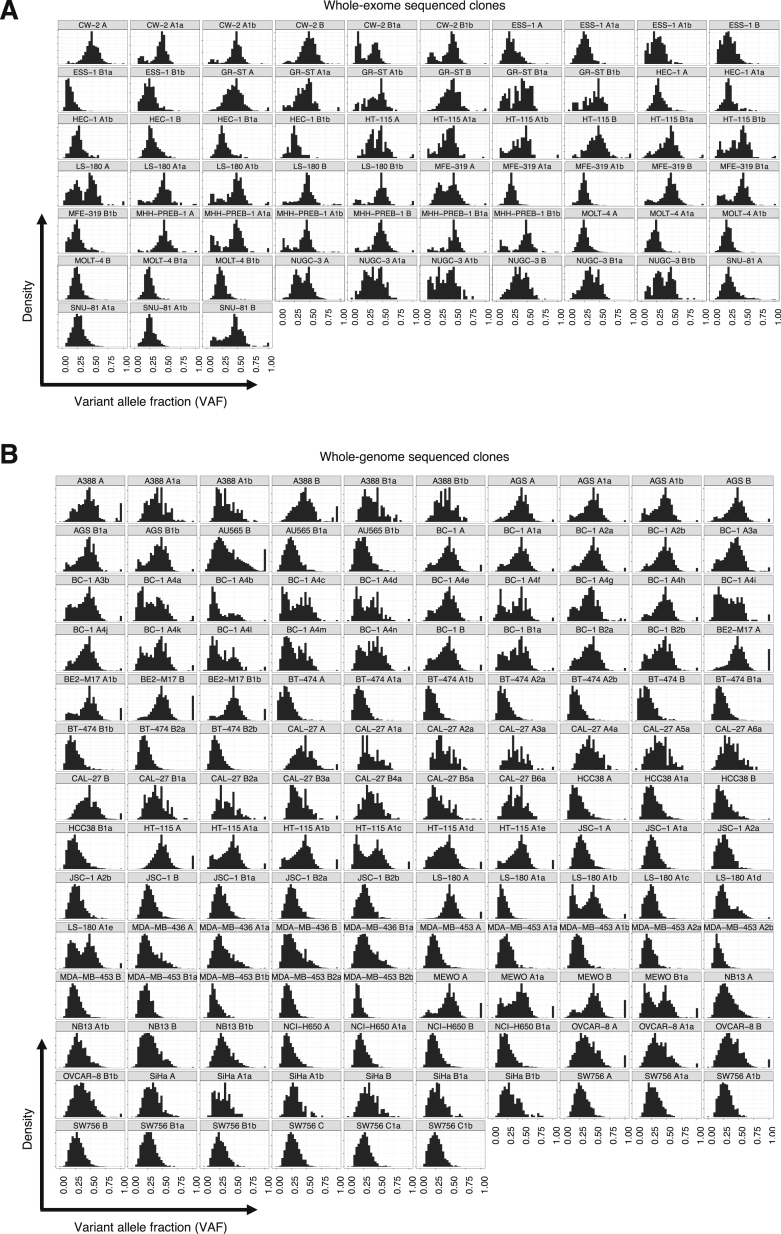
Variant Allele Fraction Distribution Plots for Cell Line Clones, Related to Figure 3, Figure 4, Figure 5 (A and B) Distribution plots showing frequencies of the variant alleles fractions (VAFs) of mutations that remain after the filtering steps (STAR Methods) in indicated clones analyzed by whole-exome (panel A) or whole-genome sequencing (panel B). VAF peaks often deviate from 50%, expected for clonal heterozygous somatic mutations in a diploid genome, because cancer cell lines are often polyploid and heterozygous copy number changes across the genome can further modulate the distribution of the VAF. Bimodal distributions and subclonal peaks can arise from mixed effects of mutations being acquired on different copy number states of the genome and/or subclonally. Minor proportion of mutations presenting at 100% of the reads in some clones can reflect loss of heterozygosity at the loci of the newly acquired mutations or residual germline variants, mainly in parent clones that were compared against the unmatched normal human genome (STAR Methods).

Using this approach, variants that were present in the most recent common ancestor cell of the related parent clones, but reside on an allele that was subsequently lost in only one of the clones would be misclassified as ‘private’. Loss of an allele in the setting of a polyploid cell line setting may not always manifest as B allele frequency ∼1 (BAF, a measure of allelic copy number ratio (Peiffer et al., 2006)), expected following the loss-of-heterozygosity (LOH) in a diploid setting. Hence, we argued that regions that vary considerably in copy number states and mutation rates between the related parent clones may exhibit allelic losses. Mutations residing within such regions were therefore removed as they could not be correctly classified as ‘shared’ or ‘private’. This approach removed majority (but not all, see Figure S7) residual germline variants. To identify regions with likely LOH, the allele frequencies of SNPs from 1000 genomes project (1000 Genomes Project Consortium et al., 2015) were obtained from whole-genome sequenced parent clones. Heterozygous SNPs were identified as those SNPs with BAF between 0.1 and 0.9, inclusive, in all related clones. For all heterozygous SNPs, BAF and logR were calculated from the ref and alt allele frequencies using the following equations:

$$BAF=\max\left(f_{ref}f_{alt}\right)/\left(f_{ref}+f_{alt}\right)$$

$$logR_{unadj}=log_{2}\left(f_{ref}+f_{alt}\right)$$

$$logR=logR_{unadj}-mean\left(logR_{unadj}\right)$$

where *f*_*ref*_ = allele frequency of 1000 genomes a0 allele, *f*_*alt*_ = allele frequency of 1000 genomes a1 allele, *logR*_*unadj*_ = unadjusted logR, *mean(logR*_*unadj*_*)* = the mean value of *logR*_*unadj*_ across all 1000 genomes loci. To identify regions with variable copy number, *logR* and BAF of related samples were jointly segmented using the *multipcf* function in the R package *copynumber* (Nilsen et al., 2012). Joint analysis of parent clones allowed the identification of both copy number aberrations shared between clones and aberrations that are unique to one sample. Regions with variable copy number states and mutation rates were identified as follows:

(i) Regions with differences in BAF of > 0.03 and < 0.01 between related clones were classified as, respectively, ‘variable’ and ‘constant’ in copy number. Regions with differences in logR outside the range between the 5^th^ and 95^th^ centiles of the difference in logR between related clones in regions of ‘constant’ copy number regions were reclassified as ‘variable’.
(ii) Regions with variable copy number states and variable mutation rates were identified as regions variable in copy number states with significant differences in the ratios of mutation rates compared to the ratio of rates calculated for regions constant in copy numbers (Poisson test p < 0.01).

#### Clonality of the cell line clones

Clonality of the established cell line clones was examined using the proportion of the mutation-reporting reads (equivalent to variant allele fraction, VAF) at the mutation loci remaining after the described filtering steps. Most of the VAF distributions deviated from average 50% (Figure S7) expected for clonal heterozygous somatic mutations occurring in a diploid genome because cancer cell lines are often polyploid and exhibit multiple copy number changes.

Some clones exhibited a shift toward lower distribution of VAF compared to other related clones, pointing to putative establishments of such clones from multiple cells or from single cells that underwent genomewide increases in the ploidy. Such instances may result in an increased mutational burden due to availability of more DNA bases and hence more opportunity for a mutation to occur (for example, see ESS-1_B1a clone with increased mutational burden and a shift toward lower VAF compared to other related clones, Figures 3 and S7). Some clones presented with bimodal VAF distributions and subclonal peaks (for example, see HT-115_A1c or BC-1 A4a-A4n, Figure S7). These may reflect subclonal mutations (which occurred during the clonal expansions of the isolated single cells) and/or clonal and subclonal mutations presenting within the regions of the genome that underwent copy number changes. Nevertheless, mutational signatures identified in such clones still point to continuing activities of the underlying processes and hence they were not removed from analysis. Furthermore, described effects are not underlying the highly fluctuant and episodic nature of observed APOBEC-associated mutagenesis. For example, although some of the MDA-MB-453, BC-1 and CAL-27 clones present with lower average VAFs compared to other clones (for example, MDA-MB-453 clones B1b, A2b, B2b; Figure S7) or with non-unimodal distributions of VAFs (for example, BC-1 clones A4a-n), many other clones from these cell lines have consistent VAF distributions and still exhibit highly fluctuant mutational rates in SBS2 and SBS 13 (for example, BC-1 A2b and A2a; CAL-27 A3-5). It is indeed possible that some of the peaks presenting at lower VAFs reflect a burden of mutations acquired following isolations of the single cells during the burst of APOBEC-associated mutagenesis. Importantly, all of the daughter and granddaughter clones from BT-474 and JSC-1 cell lines show consistent VAF distributions and highly variable SBS2 and SBS 13 mutation rates. Finally, whereas described scenarios may influence the burdens of acquired mutations, they are unlikely to do so to the extent at which fluctuations in APOBEC-associated signatures were seen (sometimes 100–fold), that are in stark contrast to other signatures detected in the same set of clones (Table S3).

#### Mutational catalogs from single cells

Whole genome amplification (WGA) of single cell DNA leads to (1) loci dropouts, (2) allele dropouts where only some alleles at the loci are amplified or (3) imbalanced amplification of alleles presenting at given loci. To estimate the proportion of somatic mutations that were likely missed due to WGA-associated losses, we annotated a set of high confidence heterozygous germline SNPs (1000 Genomes Project Consortium et al., 2012) on the sequence data from 18 stock cell lines from which single cells were derived (mean 165,000 SNPs per cell line) and assessed their representation in the sequence data from the individual cells. It was estimated that on average across all single cells examined, (1) ∼30% of the variants were lost due to loci dropouts, (2) ∼32% due to allele dropouts and (3) ∼12% of the variants presented at VAF below the optimal detection threshold (< 10%) of CaVEMan. Overall, we estimate that high-quality sequence data was generated from ∼26% of the average single cell genome. Metrics for individual cells are available in Figure S6A.

Mutations in single cells were discovered by using related stock cell lines as the reference sequences (comparisons in Table S2) and, analogous to parent clones, predominantly comprise mutations acquired between the establishment of the most recent common ancestor cell of the stock cell line and isolations of the single cells. To remove possibly pre-existing mutations acquired prior to this time frame, base substitutions were considered only if (1) the mutation loci were covered by ≥15 reads in the reference stock cell lines and (2) mutation-reporting reads were not detected in the corresponding stock cell lines.

Mutational catalogs of single cells contain a large burden of variants introduced during the isolation and/or amplification of single-cell DNA. We reasoned that the majority of such variants were here extracted in forms of SBS scE and scF (Figure 6B), characterized predominantly by C>T mutations previously associated with false positive variants arising during the WGA reactions (Leung et al., 2015). Furthermore, these signatures were detected in single cells, but absent from their corresponding stock cell lines (Figures S6) and have never been identified among the primary human cancers (Alexandrov et al., 2018). Unlike the patterns of APOBEC-associated SBS2 and SBS13, patterns of SBS scE and scF (in particular, C>T at GCN contexts) were enriched among mutations presenting at < 50% of the sequencing reads in single cells, both genomewide and when only haploid regions were considered (Figure S6B) (cell line copy number was obtained from COSMIC Cell Line Project, v.83), further supporting the proposition that these do not reflect signatures of genuine mutations. Removal of variants presenting at < 50% VAF from mutational catalogs of single cells removed a large portion of these signatures and allowed higher sensitivity in identifying SBS2 and SBS13 (see Figures 6A and S6A for annotation of mutational signatures on, respectively, filtered or complete mutational catalogs from single cells).

#### Mutational catalogs from PDX models

Mutational catalogs from PDX models were downloaded from NCI PDMR (v1.3; https://pdmr.cancer.gov/default.htm). Germline mutations were filtered out from the lists of reported mutations using the complete list of germline mutations from dbSNP (Sherry et al., 2001), 1000 genomes project (1000 Genomes Project Consortium et al., 2012), NHLBI GO Exome Sequencing Project (Fu et al., 2013), and 69 Complete Genomics panel (http://www.completegenomics.com/public-data/69-Genomes/) (Drmanac et al., 2010), as well as BAM files of (unmatched) normal tissues containing more than 120 normal genomes and 500 normal exomes, as described above.

#### Base substitution and indel classification

Base substitutions were classified into 96-channel catalogs based on their sequence context, by considering the bases immediately 5′ and 3′ to each mutated base using the ENSEMBL Core APIs for human genome build GRCh37. Because there are six classes of base substitutions (C > A, C > G, C > T, T > A, T > C, T > G; mutations are referred to by the pyrimidine of the mutated Watson–Crick base pair) and 16 possible sequence contexts for each mutated base, there are 96 possible mutated trinucleotides.

Indels were classified as described previously (Nik-Zainal et al., 2016), by interrogating the sequences at indel junctions, according to whether they were repeat-mediated, microhomology-mediated or neither. Complex indels were considered separately given the ambiguity in classification.

#### *Kataegis* quantification

*Kataegis*, or foci of localized hypermutation (Nik-Zainal et al., 2012a), were quantified in 100 whole-genome sequenced daughter clones (Figure 5). A *kataegis* focus was defined as a cluster of 5 or more consecutive mutations, which reside in APOBEC-associated sequence contexts (C>A, C>T and C>G substitutions at TCN trinucleotides), exhibiting strand-coordination and have an average intermutation distance of ≤1,500 bp. This approach may miss some foci, sacrificing sensitivity of detection in obtaining higher positive predictive value of *kataegis* foci.

#### Sequence context quantification and enrichment analysis

Sequence contexts of interest were quantified in the reference human genome (GRCh37) across autosomal chromosomes and within regions considered by the CaVEMan algorithm in detection of base substitutions (Figures S3B and S4C). The enrichment of APOBEC-like mutations within autosomal chromosomes at the quantified sequence contexts was calculated as described previously (Chan et al., 2015). Only C>T and C>G mutations were considered, as C>A mutations at APOBEC-characteristic context (TCN) frequently arise during cell cultivation. Briefly, enrichment analysis quantifies how frequently C>T and C>G mutations occur in a sequence context of interest, compared to the total number of C>T and C>G mutations. For example, to calculate enrichment for mutations at TCN sites the following was used:

$$E_{TCN}=\;\frac{Mut_{TCN}/Con_{TCN}}{Mut_{C}/Con_{C}}$$

*Mut*_*TCN*_ represents the number of TCN > TGN or TCN > TTN mutations; *Mut*_*C*_ the number of C>G and C>T mutations; *Con*_*TCN*_ and *Con*_*C*_ represent the number of occurrences of TCN contexts (and their reverse complements, NGA) and cytosines (or guanines), respectively.

#### Doubling time measurements

Doubling times were measured for a total of 26 available daughter and granddaughter clones from the BC-1, JSC-1, MDA-MB-453 and BT-474 cell lines. Clones were thawed and recovered in culture for 14-21 days prior to seeding. 100,000 cells were seeded from each clone and counted every 24 to 72 hours over the course of 8 to 15 days in triplicates. Alive cells, stained with Trypan Blue (Sigma-Aldrich), were counted with a hemocytometer. Doubling times were derived from the cell counts assuming the logarithmic growth,

$$N_{t}=N_{0}\;\times \;2^{t/T}$$

N_0_ and N_t_ are the cell counts at the seeding time and at the examined time, respectively. T is the doubling time and t are days past seeding at the examination. A linear regression was performed in R using the ‘lm’ function. The 90% confidence interval was calculated using the ‘prd’ function. Numbers of divisions which took place during the examined *in vitro* periods were estimated for each clone from the measured doubling times and durations of examined time frames (Table S2). Linear regressions between cell divisions and the burden of acquired mutational signatures for each cell line were performed using the ‘lm’ function in R.

#### Pathogen detection

To detect viral DNA sequences in the available whole-genome sequencing data from parent and daughter clones (data not shown), read-pairs which had one or both reads unmapped were identified and bases with Phred quality score < 10 were removed. The remaining sequence was split into non-overlapping 30bp fragments. Terminal fragments were processed without further splitting (30-59bp). The obtained fragments were aligned to the viral GOTTCHA database (Freitas et al., 2015), at the taxonomic levels of family, species, genus and strain using BWA (Li and Durbin, 2010). Presence of viral sequences was defined in those cases where at least 5% of the viral genome was covered with a minimum average depth of two reads across multiple clones from the same cell line. Presence of EBV was determined by examining the average read coverage and mean mapping quality across the viral genome present in the reference human genome (GRCh37) and was not detected in any of the examined cell lines, other than JSC-1 and BC-1 previously reported to carry the virus (Callahan et al., 1999, Cannon et al., 2000).

#### Identification of L1 mobile element insertions acquired *in vitro*

Non-reference L1 insertions were identified with TraFiC-mem v1.1.0 (https://gitlab.com/mobilegenomes/TraFiC), an improved version of the TraFiC (Transposon Finder in Cancer) algorithm (Rodriguez-Martin et al., 2017, Tubio et al., 2014). TraFiC-mem is based on discordant read-pair analysis as TraFiC, but it uses BWA-mem instead of RepeatMasker as a search engine for the identification of retrotransposon-like sequences in the sequencing reads and it incorporates an additional module for reconstructing the insertion breakpoints through local *de novo* assembly. TraFiC-mem was executed for a total of 100 daughter (and granddaughter) clones using the corresponding parent (and daughter) clones as reference sequences to identify L1 insertions acquired during the examined *in vitro* time frames (Table S5). Filtering of somatic L1 candidate insertions was performed following the criteria defined previously (Tubio et al., 2014), but with an additional step consisting of the removal of somatic candidates if they match a germline retrotransposition of the same family identified across > 5,000 non-cancerous genomes from the 1000 Genomes (Sudmant et al., 2015) and PCAWG projects (Campbell et al., 2017, Waszak et al., 2018). Finally, the identified L1 insertions were subject to visual inspection of BAM files with the Integrative Genomics Viewer (IGV) (Robinson et al., 2011) and removed if detected in any number of reads in the corresponding reference clones.

#### RNA sequencing and analysis

RNA was extracted using RNeasy Plus kits (QIAGEN) from 26 daughter clones (Table S2). To control for non-biological variation in expression data, ERCC Spike-In Mix (Thermo Fisher) was added (1ug total RNA spiked with 2uL of 1:100 ERCC mix). rRNA was depleted using Ribo-Zero rRNA Removal Kit (Illumina) and 150 base short-insert cDNA libraries generated using KAPA Stranded mRNA-Seq Kit (Illumina), following the balanced block design (Auer and Doerge, 2010). Sequencing (paired end, 75-bp read length) was performed on Illumina HiSeq 2500 platform. The sequencing reads were mapped using TopHat2 (Kim et al., 2013) (version 2.1.1) to the reference human genome (GRCh37) supplemented with control sequences to allow mapping of the Spike-In control RNA. Duplicate reads were removed using the Picard (MarkDuplicates) tool (version 1.60; https://broadinstitute.github.io/picard/). Gene expression levels were estimated with the default settings of the Cufflinks tool (Trapnell et al., 2010) (v.1.0.2), using a reference General Feature Format (GFF) file derived from Ensembl version 58. Only the high-confidence values (Cufflinks status *‘OK’*) were considered in derivation of FPKM values and further analyses. A positive correlation between expected and observed burden of control Spike-In RNA implied the minimal technical variation affecting the data interpretation (not shown).

#### 1,001 cell line panel expression and methylation datasets

Expression and methylation datasets from the cell line panel are available at https://www.cancerrxgene.org/gdsc1000/ and were processed previously (Iorio et al., 2016). The available expression dataset was generated using the Robust Multi-Array Average (RMA) method (Iorio et al., 2016) and it was normalized gene-wisely here to conduct analysis at the individual sample level and overcome the lack of transcriptional data from matched normal samples. The probability distribution $P_{g}$ describing the expression of a given gene g across the cell lines was estimated using a non-parametric Gaussian kernel estimator. Each expression value $x_{g,l}$ (of gene g in cell line l) was assigned a normalized expression score equal to

$$z_{g,l}=log\left(\frac{CDF_{g}\left(x_{g,l}\right)}{1-CDF_{g}\left(x_{g,l}\right)}\right),$$

where $CDF_{g}\left(x\right)$ is the value assumed by the cumulative distribution of the gene g expression at x.

### Quantification and statistical analysis

Statistical analysis was performed using indicated algorithms and tests. Graphics were produced using R version 3.2.2: A language and environment for statistical computing (R Foundation for Statistical Computing, Vienna, Austria).

#### Mutational Signatures Analysis

##### Mutational signatures identified in ICGC PCAWG Platinum release

The set of mutational signatures annotated across cell line and PDX datasets was extracted across 2,709 primary human cancers, as part of the Platinum version of ICGC PCAWG release (Alexandrov et al., 2018). The 96-channel mutational catalogs of the corresponding primary cancers were provided by PCAWG ICGC Mutational Signatures group (Table S3).

The computational framework for identification of mutational signatures across the Platinum PCAWG dataset incorporated two independent and distinct steps, termed SigProfiler (v. 2.1) and SigProfilerSingleSample (v. 1.2) (Alexandrov et al., 2018), based on previously developed methodologies (Alexandrov et al., 2015, Alexandrov et al., 2013b, Nik-Zainal et al., 2016). The code for both tools is freely available and can be downloaded from: https://www.mathworks.com/matlabcentral/fileexchange/38724-sigprofiler. The first step (SigProfiler) encompasses a hierarchical *de novo* extraction of mutational signatures based on somatic mutations and their immediate sequence context, while the second step (SigProfilerSingleSample) estimates the numbers of somatic mutations in an individual sample associated with a given set of mutational signatures. Numerical and graphical patterns of the 48 Platinum set of PCAWG signatures, including 9 signatures associated with technology-associated artifacts (termed ‘R1-9’ signatures), are provided in Figure S1 and/or Table S1. Table S3 provides the estimated numbers of somatic mutations associated with these mutational signatures in 2,709 cancer samples.

##### Framework for analysis of mutational signatures on cell line and PDX datasets

Mutational signatures were annotated on cell line and PDX datasets using SigProfiler (v.2.1) and the SigProfilerSingleSample (v.1.2), modified as described below.

##### SigProfiler hierarchical *de novo* extraction of mutational signatures

SigProfiler was first used for *de novo* discovery of mutational signatures across five separate datasets, including 96-channel mutational catalogs (Table S3) from (1) exome sequences from 1,001 human cancer cell lines, (2) exome sequences from 577 PDX models and 25 of the available originating tumors, (3) exome sequences from 63 cell line clones, (4) whole-genome sequences from 136 cell line clones and (5) whole-genome sequences from 36 single cells.

For a given set of the mutational catalogs, the previously developed algorithm (Alexandrov et al., 2013b) was applied in a hierarchical manner to an input matrix $M\;\in \;R_{+}^{K\times G}$ of non-negative natural numbers with dimension *K* × *N*, where *K* reflects the number of mutation types and *G* corresponds to the number of samples. The algorithm first deciphers the minimal set of mutational signatures that optimally explains the proportion of each mutation type and then estimates the contribution of each signature across the samples. More specifically, the algorithm makes use of a well-known blind source separation technique, termed nonnegative matrix factorization (NMF). NMF identifies the matrix of mutational signatures, $P\;\in \;R_{+}^{K\times N}$, and the matrix of the activities of these signatures, $E\;\in \;R_{+}^{N\times G}$. Identification of the unknown number of signatures, *N*, is based on the robustness of the overall solution; the methodology has been previously described (Alexandrov et al., 2013b). The identification of *M* and *P* is done by minimizing the generalized Kullback-Leibler divergence:

$$\underset{{P\in R}_{+}^{\left(K,N\right)}E\in R_{+}^{\left(N,G\right)}}{\text{min}}\sum_{ij}\left(M_{ij}log\frac{M_{ij}}{{\hat{M}}_{ij}}-M_{ij}+{\hat{M}}_{ij}\right),$$

where $\hat{M\;}\;\in \;R_{+}^{K\times G}$ is the unnormalized approximation of M, i.e., $\hat{M\;}=P\;\times \;E$. The framework is applied in a hierarchical manner to increase its ability to find mutational signatures present in few samples as well as mutational signatures exhibiting a low mutational burden. More specifically, after application to a matrix *M* containing the original samples, the accuracy for explaining the mutational spectra of each of the cancers with the extracted mutational signatures is evaluated. All samples that are well-explained by the extracted mutational signatures are removed and the framework is applied to the remaining sub-matrix of *M*.

The extracted signatures were compared to the set of mutational signatures deciphered from the PCAWG Platinum release (Table S1). Given the high proportions of germline variants in mutational catalogs from 1,001 cell lines (dataset 1) and 602 PDX models and their originating tumors (dataset 2) due to non-availability of the normal reference samples, we only considered two newly extracted signatures: SBS25 discovered in Hodgkin’s lymphoma cell lines (Figure S1; Table S1), because mutational signatures analysis was not available from primary Hodking lymphomas (Alexandrov et al., 2018); and signature termed ‘SNP signature’ (Table S1), characterized by T>C mutations at NTG context believed to reflect the residual germline variants, which commonly present as C>T mutations at CpG islands, but on rare occasions may also present as T>C variants at TpGs in the reference genome. All signatures extracted across all cell line clones (datasets 3 and 4) could be explained by a combination of signatures from the global set (cosine similarity > 0.75) and hence none were considered as novel. Signatures extracted across complete mutational catalogs from single cells (dataset 5) revealed two novel mutational signatures, termed SBS scE and scF, likely associated with the process of single cell lysis and/or WGA of single DNA molecule (see section *Mutational catalogs from single cells*).

##### Assignment of mutational signatures with SigProfilerSingleSample

The hierarchical extraction was followed by estimating the contributions of the given sets of mutational signatures in individual mutational catalogs (provided in Table S3 for all samples considered) using *SigProfilerSingleSample.* The core set of mutational signatures (Figure S1; Table S1) was annotated on all cell line and PDX samples and included 39 signatures discovered across the PCAWG Platinum dataset and SBS25, discovered in Hodgkin’s lymphoma cell lines. Additional signatures (Table S1) were annotated on specific datasets and included the SNP signature annotated on mutational catalogs from 1,001 cell lines and 602 PDX models and their originating tumors, due to high levels of germline variation in the corresponding datasets; R1 through R9 signatures, associated with technology-related artifacts (Alexandrov et al., 2018), were annotated on the PDX dataset to detect possible technology-related artifacts, because the sequencing data and corresponding quality controls were generated externally; and scE and scF signatures were annotated on mutational catalogs from single cells and their related stock cell lines.

For each examined sample, $C\;\in \;R_{+}^{K\times 1}$, the estimation algorithm consists of finding the minimum of the Frobenius norm of a constrained function (see below for constraints) for a set of vectors $S_{i=1..q}\in \;\text{Q}$, where Q is a subset (albeit, not necessarily a proper subset) of the given set of mutational signatures P, i.e., Q⊆P.

(1) $$min{\left||\vec{C}-\sum_{r=1}^{q}\left(\vec{S_{r}}\times E_{r}\right)|\right|}_{F}^{2}$$

In Equation 1,$\;\vec{C}$ and $\vec{S_{r}}$ represent vectors with *K* number of nonnegative components reflecting, respectively, the mutational catalog of a sample and the *r-th* given mutational signature. Further, both vectors have known numerical values either from the *de novo* extraction (i.e.,$\;\vec{S_{r}}$) or from generating the original mutational catalog of the sample (i.e.,$\;\vec{C}$). In contrast, $E_{r}$ corresponds to an unknown scalar reflecting the number of mutations contributed by signature $\vec{S_{r}}$ in the mutational catalog $\vec{C}$. Further, the minimization of Equation 1 is always performed under two additional constraints: (i) $E_{r}\geq 0$ and (ii) ${\left||\vec{C}|\right|}_{1}\geq E_{r}$; The constrained minimization of Equation 1 is performed using a nonlinear convex optimization programming solver using the interior-point algorithm (Byrd et al., 1999). Assignment by SigProfilerSingleSample follows a multistep process, where Equation 1 is minimized multiple times with additional constraints (see schematic description below).

First, the subsetQ contains all signatures that are found in the primary cancers (PCAWG Platinum release, see Table S3) from the matching cancer types. For this purpose, all cell line samples and PDX models were reclassified to the most closely matching types of primary cancers available from PCAWG Platinum release (as per Table S3). Any signature violating a biologically meaningful constraint (Alexandrov et al., 2018) based on a transcriptional strand bias or a total number of somatic mutations was excluded from the set Q. Further, for the 1,001 cell lines any signature violating a biologically meaningful constraints (Alexandrov et al., 2018) based on number of indels was also excluded from the set Q. Further, any $\vec{S_{r}}\times E_{r}$ for which the cosine similarity between$\;\hat{C}$ and $\vec{C}$ does not increase more than 0.01 are removed, where $\hat{C}=\sum_{r=1}^{q}\left(\vec{S_{r}}\times E_{r}\right)$. After this assignment is completed, Equation 1 is minimized by allowing all remaining signatures, i.e., $S_{i=1..q}\in \;\text{Q}\backslash \text{P}$, to be added provided that each signature increases the cosine similarity between$\;\hat{C}$ and $\vec{C}$ with more than 0.025, where $\hat{C}=\sum_{r=1}^{q}\left(\vec{S_{r}}\times E_{r}\right)$. Lastly, during all minimization steps, an additional biological condition was enforced allowing SBS1 and SBS5 in all samples to account for residual germline mutations (Rahbari et al., 2016).

#### Relationships between somatic retrotransposition and APOBEC-associated SBS2 and SBS13

The relationship between mutational signatures and somatic retrotransposition was first examined across 100 cell line daughter (and granddaughter) cell line clones. We applied generalized linear mixed model (GLMM) Poisson regression analysis to burdens of *in vitro* acquired mutational signatures and rates of L1 insertions (Tables S3 and S5, respectively). For each signature, we fitted two Poisson regression models (a null model and an alternative model) for the count of mutations assigned to the specified signature. In the null model the count of mutations *y* does not depend on the L1 insertion rate, while the alternative model incorporates the dependence. Model fitting was performed using the function glmer (R package lme4), using the Poisson family with a log link function. In the case of the null model, for each cell line experiment (*i*), the fitted model has a mean mutation count *m*_*i*_ given by $\log\left(m_{i}\right)=c+\log\left(t_{i}\right)+a\left(T_{i}\right)+e_{i}$, where *c* is a constant, *t*_*i*_ is the time spent in culture by the cells in experiment *i*, *T*_*i*_ is the tumor type from which the cell line was derived, *a(T*_*i*_*)* denotes the random effect of this tumor type factor, and *e*_*i*_ denotes a random effect which is specific to the experiment. In the case of the alternative model we introduce a predictor variable *u* which depends on the L1 insertion rate xt(where *x* is the L1 insertion count, and *t* is the time spent in culture). The predictor variable *u* is defined as u=log(1+xt). The use of this predictor variable should avoid introducing any additional dependence on the time spent in culture *t*_*i.*_ Furthermore, the log(*t*) term is an “offset” which ensures that the estimated effect sizes (including the slopes) do not depend on the time in culture. The fitted model has a mean mutation count *mi* given by $\log\left(m_{i}\right)=c+\log\left(t_{i}\right)+a\left(T_{i}\right)+e_{i}+b\left(T_{i}\right)\;u_{i}$, where *bT*_*i*_ denotes the random effect of this tumor type factor on the slope of the predictor variable *u*_*i*_, and as before, *aT*_*i*_ denotes the random (intercept) effect of this tumor type factor. The Wald test was used to test the null model against the alternative model. A separate test was performed for each signature (n = 5) and the Bonferroni procedure was applied to adjust the significance thresholds for multiple testing.

The relationship between the individual mutational signatures and the rate of somatic L1 insertions was investigated further on 2,353 primary human cancers from patients with a known age at the diagnosis project (Table S5 lists considered samples and age at diagnosis) and previously annotated both mutational signatures (Alexandrov et al., 2018; see Table S3) and somatic L1 insertions (v.1; Rodriguez-Martin et al., 2017) as part of the ICGC PCAWG, using the same GLMM Poisson regression analysis. The null model and alternative model were as previously specified, with the index variable *i* referring to an individual primary cancer, and *t*_*i*_ representing the age of the patient at diagnosis. The L1 insertion rate was defined as xt, where *x* is the L1 insertion count, and *t* is the patient age at diagnosis. A separate test was performed for each signature and the Bonferroni procedure was applied to adjust the significance thresholds for numbers of successful tests performed (i.e., numbers of signatures tested; n = 31).

### Data and software availability

Sequence data generated in this study has been deposited at the European Genome-Phenome Archive (https://www.ebi.ac.uk/ega/) with accession numbers:

Whole-exome sequencing: EGA: EGAD00001004201
Whole-genome sequencing: EGA: EGAD00001004203
RNA sequencing: EGA: EGAD00001004202

The most recent versions of our mapping and mutation calling pipelines, and supporting documentation, can be accessed from:

https://dockstore.org/containers/quay.io/wtsicgp/dockstore-cgpmap
https://dockstore.org/containers/quay.io/wtsicgp/dockstore-cgpwxs
https://dockstore.org/containers/quay.io/wtsicgp/dockstore-cgpwgs

## Supplemental Information

Table S1: Mutational Signatures Used in the Study, Related to Figures 1, 3, 5, and 6 Signatures are displayed based on the probabilities of the 96 substitution classes, defined by the substitution class and sequence context immediately 5′ and 3′ to the mutated base, on the basis of the trinucleotide frequencies of the whole human genome. These include signatures discovered in the ICGC PCAWG Platinum dataset and other signatures discovered in this study.

Table S2: Sample Sets, Data Sources, and Experimental Information, Related to Figures 1–6 List of all samples and the corresponding datasets considered in the study, including previously generated datasets (2,709 primary cancers from Platinum version of the ICGC PCAWG dataset; 1,001 cell lines from COSMIC Cell Line Project; 602 PDX models and available originating tumors from NCI PDMR) and datasets generated here (cell line clones subjected to whole-genome sequencing (WGS), whole-exome sequencing (WES) and/or RNA-sequencing; single cells and corresponding stock cell lines subjected to WGS). COSMIC cell line classification was simplified as noted, for a simpler representation in the figures.

Table S3: The 96-Channel Mutational Catalogs of All Samples and Estimated Numbers of Base Substitutions Attributed to Individual Mutational Signatures, Related to Figures 1–6

Table S4: Possibly Deleterious Aberrations in DNA Replication and Repair Mechanisms Associated with Mutational Signatures in Examined Cell Lines, Related to Figures 3 and 4

Table S5: Relationships between Mutational Signatures and L1 Retrotransposon Insertions, Related to Figures 4–5 These were examined on available whole-genome sequenced datasets, including 100 cell line daughter/granddaughter clones and 2,353 PCAWG primary cancers. Analysis was performed on complete datasets as listed in Table S2, although only those cell line samples where newly acquired retrotransposon events were detected are displayed.
